# Evolutionary Dynamics of Nitrogen Fixation in the Legume–Rhizobia Symbiosis

**DOI:** 10.1371/journal.pone.0093670

**Published:** 2014-04-01

**Authors:** Hironori Fujita, Seishiro Aoki, Masayoshi Kawaguchi

**Affiliations:** 1 Division of Symbiotic Systems, National Institute for Basic Biology, National Institute for Natural Sciences, Okazaki, Japan; 2 Department of General Systems Studies, Graduate School of Arts and Sciences, University of Tokyo, Komaba, Meguro-ku, Tokyo, Japan; 3 Department of Basic Biology, School of Life Science, Graduate University for Advanced Studies (SOKENDAI), Okazaki, Japan; Dowling College, United States of America

## Abstract

The stabilization of host–symbiont mutualism against the emergence of parasitic individuals is pivotal to the evolution of cooperation. One of the most famous symbioses occurs between legumes and their colonizing rhizobia, in which rhizobia extract nutrients (or benefits) from legume plants while supplying them with nitrogen resources produced by nitrogen fixation (or costs). Natural environments, however, are widely populated by ineffective rhizobia that extract benefits without paying costs and thus proliferate more efficiently than nitrogen-fixing cooperators. How and why this mutualism becomes stabilized and evolutionarily persists has been extensively discussed. To better understand the evolutionary dynamics of this symbiosis system, we construct a simple model based on the continuous snowdrift game with multiple interacting players. We investigate the model using adaptive dynamics and numerical simulations. We find that symbiotic evolution depends on the cost–benefit balance, and that cheaters widely emerge when the cost and benefit are similar in strength. In this scenario, the persistence of the symbiotic system is compatible with the presence of cheaters. This result suggests that the symbiotic relationship is robust to the emergence of cheaters, and may explain the prevalence of cheating rhizobia in nature. In addition, various stabilizing mechanisms, such as partner fidelity feedback, partner choice, and host sanction, can reinforce the symbiotic relationship by affecting the fitness of symbionts in various ways. This result suggests that the symbiotic relationship is cooperatively stabilized by various mechanisms. In addition, mixed nodule populations are thought to encourage cheater emergence, but our model predicts that, in certain situations, cheaters can disappear from such populations. These findings provide a theoretical basis of the evolutionary dynamics of legume–rhizobia symbioses, which is extendable to other single-host, multiple-colonizer systems.

## Introduction

Symbiosis is an ecological interaction in which two or more species exchange mutual benefits. This cooperative interaction promotes the fitness of both species, and thereby reinforces their symbiotic relationship. However, symbiotic systems are vulnerable to emerging selfish cheaters that extract benefits from the system without paying costs. Such parasitic cheaters may potentially disrupt the symbiotic relationship.

One of the well-known symbioses occurs between legume plants and rhizobia (nitrogen-fixing soil bacteria). Rhizobia establish symbiotic organs termed root nodules on the roots of their host, and proliferate by extracting nutrients from the host plant. In turn, they supply their host plants with nitrogen resources produced by nitrogen gas fixation. This mutual nutrient exchange should promote the fitness of both organisms and thereby strengthen the symbiotic relationship. This beneficial effect, known as “partner fidelity feedback”, is assumed as a stabilizing factor for the mutualistic relationship [1]–[4].

On the other hand, naturally occurring rhizobium strains vary in their nitrogen fixation activity, and ineffective rhizobia that colonize their host plants without undertaking nitrogen fixation in their root nodules are ubiquitous [5]–[8]. Because the nitrogen fixation reaction consumes much energy (or costs), such parasitic cheaters could use surplus energy for their own growth or for synthesizing storage substances. Consequently, they are likely to proliferate more efficiently than nitrogen-fixing cooperators, posing a risk to the symbiotic interaction. Rhizobia are therefore exposed to two opposite effects that simultaneously promote (by providing benefit) and destabilize (by incurring cost) the mutualistic relationship. In this paper, we refer to these effects as the “promoting force” and “destabilizing force”, respectively.

Despite the widespread presence of ineffective rhizobia, the legume–rhizobia symbiosis is evolutionarily stable. Potential mechanisms that stabilize the symbiotic interaction include partner fidelity feedback [1]–[4], partner choice [4], [9]–[12], host sanction [4], [10], [13]–[16], spatial structure [17], and kin selection (or inclusive fitness) [18]. Partner choice and host sanction exert similar effects, in that host plants discriminate beneficial symbionts. However, partner choice is often regarded as a selection process based on recognition signals, while in host sanction, plants punish more parasitic cheaters by reducing nutrient supply based on their symbiotic performance [3], [4], [10]. Thus, in this paper, we consider partner choice and host sanction as pre-infection and post-infection processes, respectively, according to the nitrogen fixation activity of symbionts.

The evolution of cooperation has been theoretically modeled by game theory, in particular, the prisoner's dilemma game and the snowdrift game (also known as the hawk-dove game) [19], [20]. If the game involves two players, mutual cheating is an evolutionarily stable strategy (ESS) in the prisoner's dilemma, while the snowdrift game has the Nash equilibrium of mixed-strategy, in which cooperator and cheater stably coexist. These games have been extensively studied under various conditions. However, although the simplified framework of binary strategy choice (full cooperation or full defection) captures the essential nature of cooperation, evolutionary dynamics involving quantitative traits (such as nitrogen fixation activity in rhizobia) require more complex frameworks. Continuous strategies, such as the continuous versions of snowdrift and prisoner's dilemma, more accurately reflect real-world phenomena [20]–[27]. Interestingly, the continuous snowdrift game permits an evolutionary process in which completely non-productive cheaters coexist with cooperators making maximum investment [23]. This evidence provides insight into the evolutionary origin of ineffective symbionts in the legume–rhizobia mutualism.

Various mathematical models have been proposed for the evolution of the legume–rhizobia symbiosis. Akcay et al. [28], [29] developed models based on bargaining theory, in which legumes and nitrogen-fixing nodule(s) negotiate to decide whether and how to form or terminate the interaction. Other models describe the population dynamics of rhizobia strains with fixed nitrogen fixation activity, such as complete cooperators or total cheaters [20], [30]–[33]. These models can explain the coexistence of cooperators and cheaters; however, as described above, discrete strategies oversimplify the evolutionary dynamics of quantitative phenotypes. On the other hand, West et al. [14], [18] reported models in which the optimal strategy (or the maximum fitness) is determined by continuously varying the nitrogen fixation. While finding the optimal strategy is a powerful approach for analyzing evolutionary systems, it cannot sufficiently explain relatively complicated situations, such as frequency-dependent interactions between different rhizobia strains. In fact, the stable co-existence of cooperators and cheaters is difficult to explain using these approaches.

In contrast, the legume–rhizobia mutualism is more directly reflected by evolutionary dynamics in which rhizobia proliferate according to the quantitative trait of nitrogen fixation with a frequency-dependent selection. However, this situation has yet to be fully investigated, although such a model may explain the effect of mixed colonization [34]. Therefore, to theoretically understand the evolutionary dynamics of the legume/rhizobium system, we construct a simple model based on the continuous snowdrift game with interacting multiple players [20], [23], and investigate the model using adaptive dynamics and numerical simulations. The aim of this paper is not to examine the nature or evolution of mechanisms that prevent rhizobia from cheating, but rather to investigate the conditions under which mutualistic and cheating symbionts can coexist. We also aim to understand how and why symbiosis establishes and stably persists despite the ubiquitous distribution of cheating rhizobia.

## Models

### 2.1 Model Framework

#### 2.1.1 Fitness function

In the legume–rhizobia symbiosis, host plants cannot extract benefits (i.e. nitrogen fixation products) without paying costs (i.e. nutrients or energy) to rhizobia, because the symbionts are confined in root nodules and thereby cannot directly interact with the external environment. This fact suggests that host plants cannot develop a cheating strategy, whereas ineffective rhizobia are ubiquitously distributed in nature. Thus, the evolutionary dynamics of the mutualism are most likely driven by the strategies of the symbionts, while the host plants play a minor role. Accordingly, our model is based on the strategies adopted by the rhizobia.

**Figure 1:**
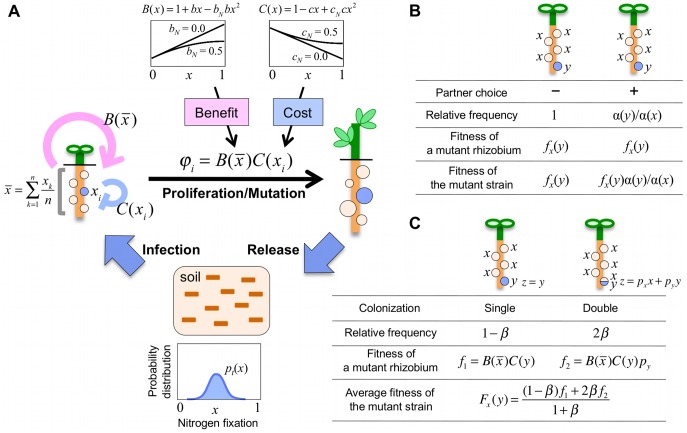
Schematic representation of model framework. **(A)** The evolutionary strategy of an individual rhizobium is its nitrogen fixation activity (0≤*x*≤1). Rhizobia stochastically inhabit host plants according to *p_t_*(*x*), the probability distribution of their strategy, proliferate in root nodules in a frequency-dependent manner according to Eq. (1), change in their nitrogen fixation ability by mutation, and are released back to the soil following the death of their host plants (Eq. (4)). Proliferation of colonized rhizobia is driven by the benefit (promoting force) and cost (destabilizing force), which depend on the nitrogen fixation activities of the rhizobia. The cost of a single rhizobium is affected by its own strategy *x_i_* (i.e. *C*(*x_i_*)) while the benefit is affected by 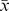: the average strategy of *n* colonizers (i.e. 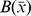). See text for details. **(B and C)** Effects of partner choice (B) and mixed nodule (C) on the fitness of rare mutants with nitrogen fixation activity *y* in the resident population with *x*. *α*(*x*) is the probability that a rhizobium with *x* is accepted by a host plant, and *β* is the frequency of nodules inhabited by two rhizobium species. Yellow and blue root nodules are colonized by residents with *x* and rare mutants with *y*, respectively.

The evolutionary strategy of each rhizobium is its nitrogen fixation activity in a root nodule, which is continuous between 0 (no activity or full defection) and 1 (maximum activity or full cooperation) and which is genetically transmitted to its progeny. Each host plant is assumed to be colonized by a constant number *n* of rhizobia, which correspondingly form *n* root nodules (Figure 1A). Thus, each root nodule harbors a clonal monomorphic population of rhizobia, except in the mixed-population model of section 3.8, where each nodule harbors one or more than one rhizobial strain. In addition, host plants are assumed to be stochastically infected with rhizobia independent of their nitrogen fixation activity (or strategy), except in the partner choice model of section 3.7, where host plants select preferential symbionts as colonists.

Because rhizobia are considered to grow much more efficiently in root nodules than in the soil, we consider only their proliferation in root nodules. Proliferation is linked to the benefit (or promoting force) and the cost (or destabilizing force) of nitrogen fixation activity. The benefit encourages cooperative growth between the host plant and its symbionts. Now consider that a host plant is infected with *n* rhizobia of nitrogen fixation activity *x_k_* (*k* = 1, 2, ····, *n*) according to the probability distribution of rhizobia in the soil, and subsequently generates *n* corresponding root nodules. The productivity of this focal plant (*φ_p_*) is promoted by the total nitrogen resource supplied by the root nodules, represented by the average of their strategies: 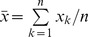, and is thus an increasing function of 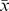: 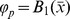. Each colonizing rhizobium (or its corresponding root nodule) in turn grows more efficiently as the host plant productivity *φ_p_* increases, Thus rhizobial growth rate (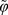) is an increasing function of *φ_p_*: 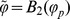, and is consequently expressed as 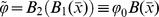 and *B*(0) = 1, where *φ*_0_ is the growth rate in the absence of nitrogen fixation (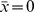) and the benefit function *B*(*x*) is an increasing function of *x*.

On the other hand, the cost (or destabilizing force) exerts an inhibitory effect on symbiont fitness, such that the growth rate of each infecting rhizobium (or its corresponding root nodule) decreases as its own nitrogen fixation activity (*x_i_*) increases; 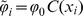 and *C*(0) = 1, where the cost function *C*(*x*) is a decreasing function of *x*. Therefore, when benefit and cost compete, the growth rate of a focal infecting rhizobium adopting strategy *x_i_* can be expressed as 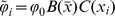. The transformation of 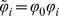 yields the relative fitness:
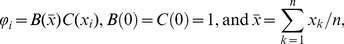
(1)where the benefit and cost functions *B*(*x*) and *C*(*x*) are strictly increasing and decreasing functions of *x*, respectively, (i.e. *B*′(*x*)>0 and *C*′(*x*)<0).

Because the promoting and destabilizing forces affect rhizobia proliferation in a synergistic rather than an additional manner, we define the fitness as the product of the benefit and cost functions (Eq. (1)). This form differs from that of conventional game theory, in which the cost is subtracted from the benefit. However, qualitatively, both forms of the fitness function yield the same evolutionary dynamics of the symbiotic system, because the evolutionary consequences are similar in the two models; namely, full defection, full cooperation, partial cooperation, and coexistence of cooperators and defectors created by “evolutionary branching” (corresponding to cases (i), (ii)/(vi), (iii), and (iv)/(v), respectively, in this paper) [23]. Consequently our model is equivalent to the continuous snowdrift game with *n* interacting players [20], [23].

#### 2.1.2 Benefit and cost functions

The benefit and cost functions are simply defined as:
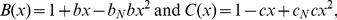
(2)where *b* and *b_N_* correspond to the strength and nonlinearity, respectively, of the benefit, and *c* and *c_N_* are the corresponding parameters of the cost (Figure 1A). These functions require that *B*(*x*)≥0, *B*′(*x*)≥0, *C*(*x*)≥0, and *C*′(*x*)≤0, which are satisfied when 0≤*b*, 0≤*c*≤1, 0≤*b_N_*≤1/2, and 0≤*c_N_*≤1/2. In this paper, *C*(*x*) is not regarded as an increasing function of *x*, because nitrogen fixation obviously strengthens if both the benefit and cost functions are increasing functions of *x* (see Text S2).

Partner fidelity feedback and host sanction are widely-discussed stabilizers of symbiosis [2]–[4], [35]–[37], and are incorporated into the benefit and cost functions, respectively, in our model. The effect of the benefit (*b*) increases as partner fidelity feedback strengthens, while the cost (*c*) decreases with increasing host sanction.

### 2.2 Numerical Calculations

In the numerical calculations, we first introduce the discrete probability distribution of the rhizobia strategies in the *t*-th cycle:
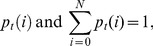
(3)where *i* (0, 1, ····, *N*) corresponds to strategy *x* = *i*/*N*. The initial distribution is a population of non-fixing rhizobia: *p*_0_(0) = 1 and *p*_0_(*i*) = 0 for *i*≠0. This strategy distribution is iteratively changed in the infection, proliferation/mutation, and release steps (Figure 1A). We consider a total number *M* of host plants, each infected with *n* rhizobia randomly selected from the probability distribution *p_t_*(*i*). We denote the probability distribution of the *M*×*n* infecting rhizobia by 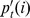. In each corresponding root nodule, the infecting rhizobia proliferate at a rate calculated by Eq. (1) and their nitrogen fixation ability is altered from strategy *j*/*N* to *i*/*N* by mutation at transition rate *μ_ji_*. Thus the probability distribution after the proliferation/mutation step can be described as a time-discretized form of the replicator–mutator equation:
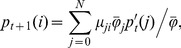
(4)where 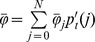 is the average fitness of the infecting rhizobia, and 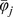 is the fitness of strategy *j*/*N*. In this paper, mutation rates are given by
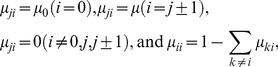
(5)where *μ* specifies the mutation rate between neighboring strategies, and *μ*_0_ specifies the rate of the null mutation. Parameters are set to *N* = 200, *M* = 2000, *μ* = 0.04, and *μ*_0_ = 0.0 or 0.01.

## Results

By investigating our model, we hope to understand the evolutionary consequences of the legume–rhizobia symbiosis in terms of adaptive dynamics (Text S1) [38], [39]. Adaptive dynamics assumes that strategy changes induced by mutations are small. However, this assumption may not hold in legume–rhizobia symbiosis, because a defect in a single gene can impart the null mutation that is responsible for complete lack of nitrogen fixation activity. Thus, we first analyze the model assuming that mutations exert little effect, and afterwards examine the impact of null mutations on the symbiotic relationship.

In our model, a host legume plant is infected with a constant number *n* of rhizobia, each of which adopts a nitrogen fixation activity 0≤*x*≤1 as its evolutionary strategy (Figure 1A). Now consider the invasibility of mutants with nitrogen fixation activity *y* in the resident population with *x*. If the mutant is rare, the probability that a host plant is colonized by multiple mutants is very low and can be neglected. Thus, assuming that a host plant is colonized by a single mutant with *y* and by *n* – 1 residents with *x*, Eq. (1) gives the fitness of the mutant strain as
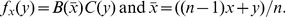
(6)


Note that, as described in section 2.1.1, the fitness is the product of the benefit and cost functions, unlike conventional game theory, in which these quantities are subtracted. We now introduce the relative fitness of the mutant to the resident:
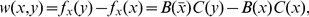
(7)which determines the invasibility of the mutant into the resident population. If *w*(*x*,*y*)>0, the mutant can invade the population, but cannot if *w*(*x*,*y*)<0. The theoretical basis of adaptive dynamics is described in Text S1.

Because legumes and rhizobia provide mutually-enhancing nutrients to each other, thereby increasing the fitness of both, the benefit function *B*(*x*) should be a strictly increasing function of *x* (i.e. *B*′(*x*)>0). In contrast, the cost function *C*(*x*) is not necessarily a simple decreasing function of *x* because it may be affected by partner choice and host sanction. If such stabilizing effects are sufficiently strong that *C*(*x*) becomes an increasing function of *x*, the maximum activity of nitrogen fixation (*x* = 1) is predicted to evolve and be stably maintained (see Text S2). Accordingly, we assume that *C*(*x*) is a decreasing function of *x* (i.e. *C*′(*x*)<0) in this paper. Note that under this condition, more cheating rhizobia proliferate more efficiently when they inhabit the same plant.

### 3.1 Linear Cost Function

In the linear cost function (i.e. *c_N_* = 0), the selection gradient 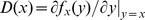 is a decreasing function of *x* (i.e. *D*′(*x*)<0 for 0<*x*<1). Accordingly the evolutionary behavior is classified into the following three cases depending on the cost–benefit balance (for details see Text S3).

#### 3.1.1 Case (i) “No evolution”

**Figure 2:**
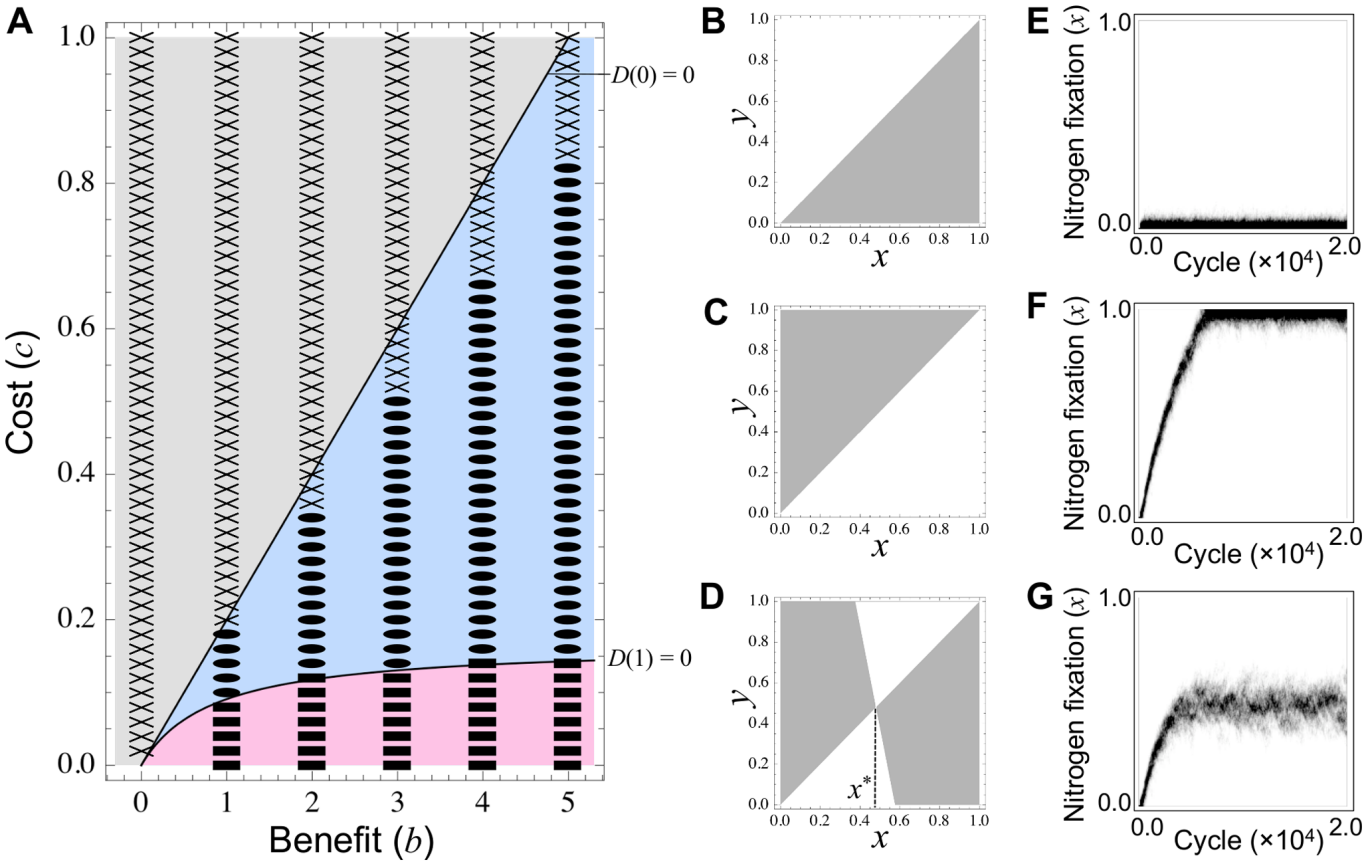
Effect of benefit and cost, assuming linear cost function. **(A)** Theoretically, the linear cost function (*c_N_* = 0) yields three evolutionary outcomes: (i) “No evolution” (gray), (ii) “Maximum evolution” (magenta), and (iii) “Intermediate evolution” (blue). This prediction is consistent with numerical simulations: cases (i), (ii) and (iii) correspond to crosses, squares and circles, respectively. **(B–D)** Pairwise invasibility plots in (B) case (i), (C) case (ii), and (D) case (iii). Rare mutants with strategy *y* can invade the resident population with *x* in the gray region (i.e. *w*(*x*,*y*)>0), but cannot in the white region (i.e. *w*(*x*,*y*)<0). **(E–G)** Evolutionary dynamics of strategy distribution in (E) case (i), (F) case (ii), and (G) case (iii); darker shades indicate higher frequencies of a strategy. Parameters are: *b* = 3.0 (B–G), *b_N_* = 0.0, *c* = 0.7 (B and E), 0.1 (C and F), and 0.22 (D and G), *c_N_* = 0.0, *n* = 5.

If *D*(0)<0 (i.e. *c*>*c*_0_ = *b*/*n*), we have *D*(*x*)<0 for 0<*x*<1 (Figures 2A and S1, gray). In this scenario, a resident population with nitrogen fixation activity *x* is always invaded by lower-performing mutants (*y*<*x*) but not by those with higher activities (*y*>*x*) (Figure 2B), and a symbiotic relationship never evolves. We refer to this scenario as the “No evolution” case.

#### 3.1.2 Case (ii) “Maximum evolution”

Conversely, if *D*(1)>0 (i.e. 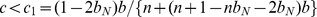) (Figures 2A and S1, magenta), we have *D*(*x*)>0 for 0<*x*<1. In this scenario, the nitrogen fixation of the resident population always increases, because only mutants with higher strategies can invade and subsequently replace the resident (Figure 2C). Thereby, the population evolves toward the maximum nitrogen fixation of *x* = 1. We refer to this scenario as the “Maximum evolution” case. A population that has evolved to *x* = 1 cannot be invaded by cheating bacteria (*x* = 0) possessing the null mutation (because *w*(1,0)<0; see Text S3.4).

#### 3.1.3 Case (iii) “Intermediate evolution”

The intermediate condition between cases (i) and (ii) (i.e. *c*_1_<*c*<*c*_0_) (Figures 2A and S1, blue), permits a unique singular strategy 0<*x* ^*^<1 that is always convergence stable (CS) and ESS-stable (i.e. *D*′(*x* ^*^)<0 and 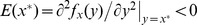) (Figure 2D) (see Text S3.3). Thus a monomorphic population should converge towards *x* ^*^ and become stably maintained with no invasion of nearby mutants. In addition, similar to case (ii), a population that has evolved to *x* ^*^ cannot be invaded by cheaters possessing the null mutation (because *w*(*x* ^*^,0)<0; see Text S3.4). This scenario is called the “Intermediate evolution” case.

The theoretical analysis described above is consistent with numerical simulations, in which an initial population of non-fixing rhizobia (*x* = 0) falls into one of three evolutionary outcomes: case (i) “No evolution” when the cost (*c*) is relatively stronger than the benefit (*b*) (Figure 2A, crosses and 2E), case (ii) “Maximum evolution” when the benefit (*b*) is relatively stronger than the cost (*c*) (Figure 2A, squares and 2F), and case (iii) “Intermediate evolution” when the benefit and cost are balanced (Figure 2A, circles and 2G).

#### 3.1.4 Speed of the symbiosis evolution

**Figure 3:**
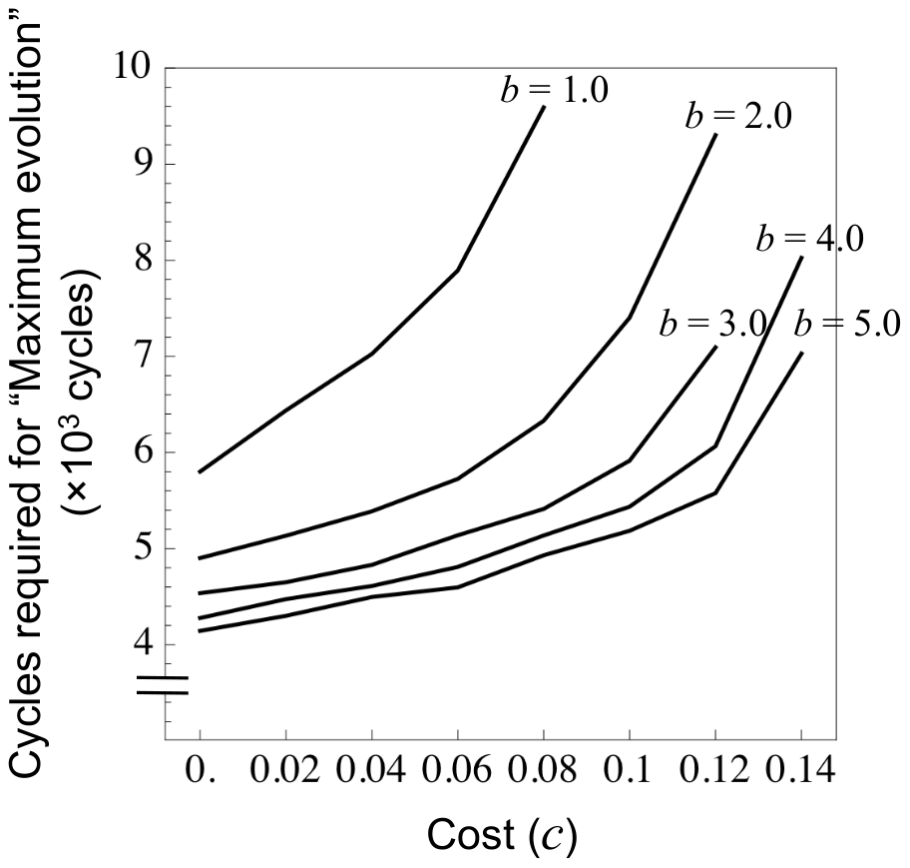
Evolution speed of symbiosis establishment. In case (ii) “Maximum evolution”, an initial population of non-fixing rhizobia (*x* = 0) evolves to one containing full cooperators (*x* = 1). The speed of this evolution increases (i.e. cycles required for “Maximum evolution” decrease), as the benefit (*b*) increases or as the cost (*c*) reduces. Each data point is the average of five calculations. Parameters are: *b_N_* = 0.0, *c_N_* = 0.0, *n* = 5.

In case (ii), an initial population of non-fixing rhizobia (*x* = 0) evolves to the maximum nitrogen fixation (*x* = 1) (Figure 2F). The evolutionary speed at which the symbiosis establishes is also affected by the cost–benefit balance. The lower the cost (or greater the benefit), the faster the evolution towards a cooperative behavior (Figure 3).

#### 3.1.5 Effect of the nonlinearity of the benefit function

Nonlinearity of the benefit function (*b_N_*) does not qualitatively affect evolutionary behaviors, but influences the parameter conditions such that, as *b_N_* increases, the parameter region of case (ii) reduces (Figure S1, magenta) while that of case (iii) expands (blue). This result signifies an inhibitory effect of *b_N_*.

### 3.2 Linear Benefit Function

**Figure 4:**
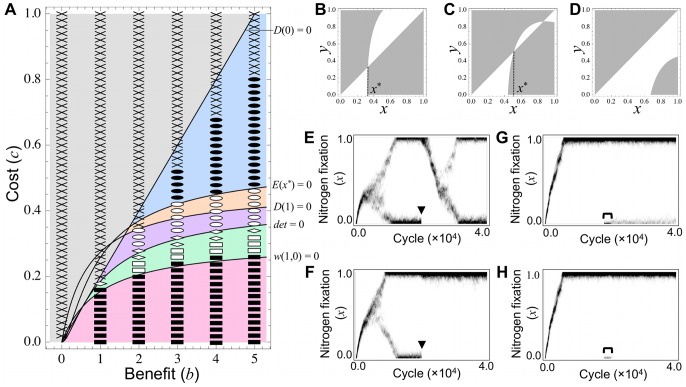
Effect of the benefit and cost, assuming a linear benefit function. **(A)** Theoretically, the benefit function (*b_N_* = 0) yields six evolutionary outcomes: (i) “No evolution” (gray), (ii) “Maximum evolution” (magenta), (iii) “Intermediate evolution” (blue), (iv) “Co-dependent coexistence” (orange), (v) “Parasitic coexistence by evolutionary branching” (purple), and (vi) “Parasitic coexistence by null mutation” (green) (for derails see Text S4). This prediction is consistent with numerical simulations; crosses, squares, closed circles, open circles, diamonds, and open squares correspond to cases (i), (ii), (iii), (iv), (v) and (vi), respectively. **(B–D)** Pairwise invasibility plots of (B) case (iv), (C) case (v), and (D) case (vi). Rare mutants with strategy *y* can invade the resident population with *x* in the gray region (i.e. *w*(*x*,*y*)>0), but cannot in the white region (i.e. *w*(*x*,*y*)<0). **(E–H)** Evolutionary dynamics of strategy distribution in (E) case (iv), (F) case (v), (G) case (vi), and (H) case (ii); darker shades indicate higher frequencies of a strategy. Once cheating bacteria with *x*<0.2 are removed from the coexistence situation (arrowheads), the remaining nitrogen-fixing bacteria can persist stably in case (v), but lose their activities in case (iv). A population of nitrogen-fixing rhizobia can be invaded by cheaters carrying the null mutation (brackets) in case (vi), but not in case (ii). Parameters: *b* = 5.0 (B–H), *c* = 0.43 (B), 0.37 (C), 0.3 (D), 0.42 (E), 0.34 (F), 0.28 (G), and 0.26 (H). In all cases, *b_N_* = 0.0, *c_N_* = 0.35, and *n* = 5.

Next, we impose a linear benefit function, namely, *b_N_* = 0. Similar to section 3.1, three cases of cooperative behavior emerge; (i) “No evolution” (Figures 4A and S2, gray), (ii) “Maximum evolution” (magenta), and (iii) “Intermediate evolution” (blue) (for details see Text S4). This model admits an intriguing outcome whereby nitrogen-fixing and cheating rhizobia coexist under the intermediate condition between cases (ii) and (iii) (orange, purple, and green). The remainder of this section is dedicated to this phenomenon. Coexistence emerges via two pathways: “evolutionary branching” (orange and purple) and null mutation (green). Furthermore, the emergent cheating rhizobia are divided into two types depending on their relationship to the symbiosis system: “co-dependent” (orange) and “parasitic” (purple and green), in which nitrogen-fixing rhizobia (or the symbiotic system) are stabilized by “co-dependent” cheaters, but persist independently of “parasitic” cheaters. Thus, coexistence can be classified into the following three cases (for details see Text S4).

#### 3.2.1 Case (iv) “Co-dependent coexistence”

In the orange parameter area in Figures 4A and S2, rhizobia adopt a singular strategy 0<*x* ^*^<1, which is CS but not ESS-stable (i.e. *D*′(*x* ^*^)<0 and *E*(*x* ^*^)>0) (Figure 4B). This situation is known to induce “evolutionary branching” or sympatric speciation, in which the monomorphic *x* ^*^ population is invaded by nearby mutants and subsequently splits into two subpopulations with higher and lower activities of nitrogen fixation than the original *x* ^*^.

This theoretical prediction is confirmed by numerical simulations, in which an initial population with *x* = 0 evolves to the singular strategy (*x* ^*^) and then splits into two subpopulations (Figure 4A, open circles and 4E). These subpopulations gradually diverge until one attains the maximum (*x* = 1; full cooperator) while the other reduces to the minimum (*x* = 0; full cheater) under most of the investigated parameter conditions. Consequently, nitrogen-fixing and cheating bacteria evolve to stably coexist.

This coexistence is predicted to be a co-dependent relationship, in which cooperators and cheaters require each other for their own survival because each monomorphic population is invaded by nearby mutants (i.e. *D*(0)>0 and *D*(1)<0; see Text S4). This prediction is also realized in numerical simulations. When cheating rhizobia are completely removed from the stable co-existence (Figure 4E, arrowhead), the remaining nitrogen-fixing bacteria lose their activities and their population destabilizes. This result suggests that cheating rhizobia stabilize the symbiotic relationship. Accordingly we name this type of coexistence “Co-dependent coexistence”.

#### 3.2.2 Case (v) “Parasitic coexistence caused by evolutionary branching”

In the purple parameter area in Figures 4A and S2, a singular strategy 0<*x* ^*^<1 exists that is CS but not ESS-stable (Figure 4C), similar to case (iv). Accordingly, cooperators and cheaters can co-evolve through evolutionary branching. However, unlike case (iv), cooperators should persist regardless of the presence or absence of cheaters (because *D*(1)≥0). This prediction is also confirmed by numerical simulations, in which cooperator bacteria can stably exist even after removal of the cheaters (Figure 4A, diamonds and 4F). In this case, the symbiotic relationship can persist independently of the existence of cheating rhizobia. We refer to this type of coexistence as “Parasitic coexistence”.

#### 3.2.3 Case (vi) “Parasitic coexistence caused by null mutation”

In the green parameter region in Figures 4A and S2, the maximum strategy will evolve similarly to case (ii) (because *D*(*x*)>0 for 0<*x*<1). However, unlike case (ii), the evolved population of nitrogen-fixing symbionts (*x* = 1) will likely be invaded by cheaters (*y* = 0) possessing the null mutation (because *w*(1,0)>0) (Figure 4D).

This type of coexistence is also confirmed by numerical simulations. Nitrogen-fixing rhizobia first evolve as in case (ii), followed by transient introduction of a null mutation, which produces cheating rhizobia (Figure 4A, open squares and 4G). To investigate the fate of the cheaters existing in the population, we then remove this mutation. As theoretically predicted, cheating rhizobia can stably persist after ceasing the null mutation under the parameter conditions of case (vi). By contrast, under the conditions of case (ii), cheaters disappear immediately after mutation removal (Figure 4A, filled squares and 4H).

#### 3.2.4 Effect of the nonlinearity of the cost function

Nonlinearity in the cost function (*c_N_*) promotes symbiosis evolution, because the parameter regions of case (ii) and (iii) increase and decrease, respectively, as *c_N_* increases (see Figure S2, blue and magenta, respectively). Furthermore, *c_N_* induces the emergence of cheaters, because the parameter area in which cheaters can stably exist (cases (iv)–(vi)) is absent under weak nonlinearity of 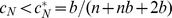, but extends with increasing *c_N_* (Figure S2, orange, purple, and green).

### 3.3 Nonlinear Benefit and Cost Functions

**Figure 5:**
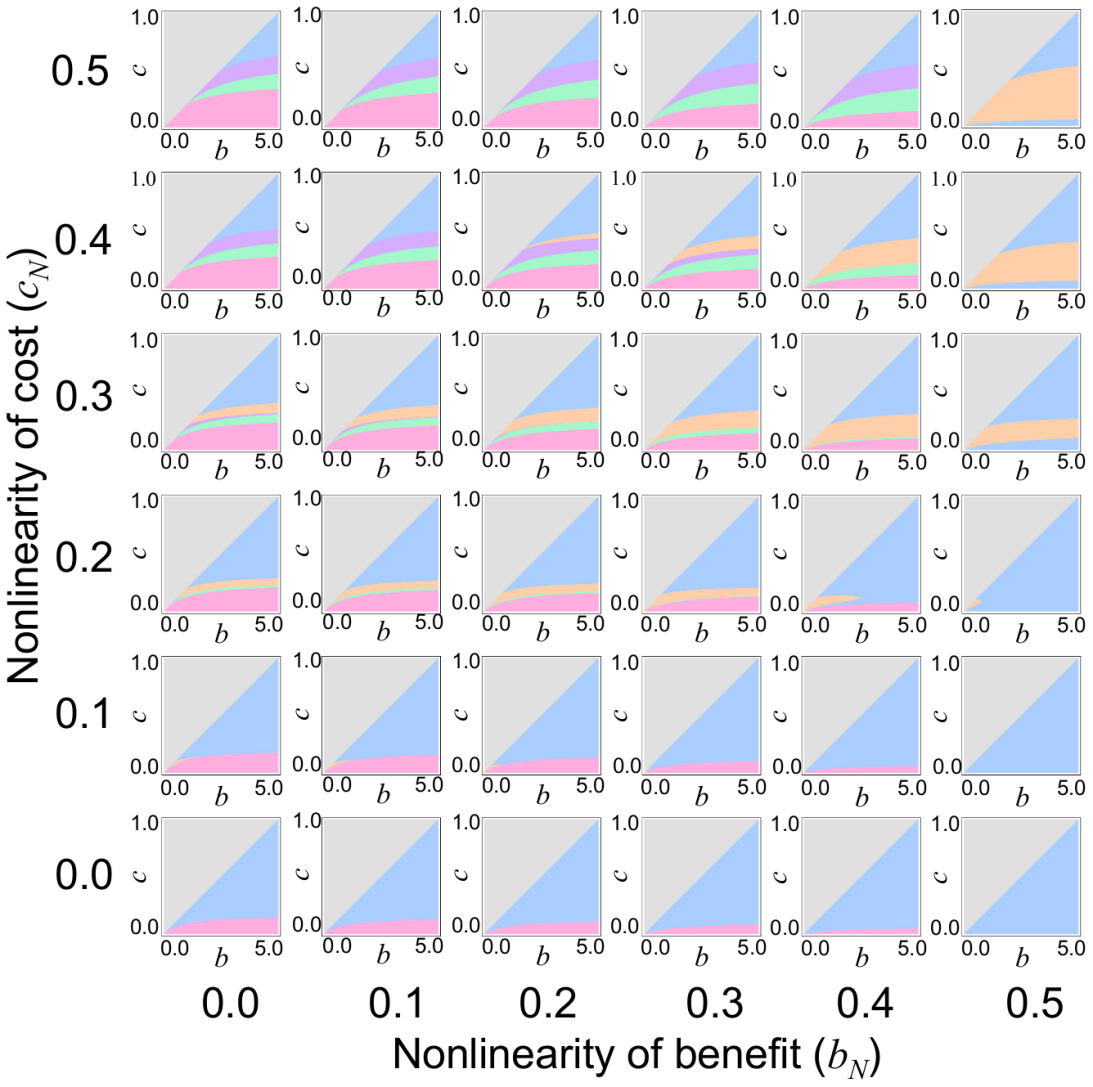
Effects of nonlinear benefit and cost functions. Nonlinear benefit and cost functions yield the evolutionary outcomes (i) “No evolution” (gray), (ii) “Maximum evolution” (magenta), (iii) “Intermediate evolution” (blue), (iv) “Co-dependent coexistence” (orange), (v) “Parasitic coexistence by evolutionary branching” (purple), and (vi) “Parasitic coexistence by null mutation” (green). Parameter regions of these cases are delineated by the properties of their singular strategy (similar to Text S4); case (i) *D*(0)<0, case (ii) *D*(*x*)>0 for 0<*x*<1 and *w*(1,0)<0, case (vi) *D*(*x*)>0 for 0<*x*<1 and *w*(1,0)>0, case (iii) x^*^ is CS and ESS-stable (i.e. *D*′(x^*^)<0 and *E*(x^*^)<0), case (iv) x^*^ is CS but not ESS-stable (i.e. *D*′(x^*^)<0 and *E*(x^*^)>0) and an unstable monomorphic population of cooperators exists (i.e. *D*(1)<0), and case (v) if x^*^ is CS but not ESS-stable (i.e. *D*′(x^*^)<0 and *E*(x^*^)>0) and a stable monomorphic population of cooperators exists (i.e. *D*(1)>0), where x^*^ is the smallest singular strategy (*D*(x^*^) = 0 and 0<x^*^<1). In all cases, *n* = 5.

Finally, we examine the effects of nonlinear benefit and cost functions. Similar to section 3.2, numerical calculation yields six evolutionary behaviors depending on the properties of the singular strategy. Under this generalized condition, the results are similar to those obtained in sections 3.1 and 3.2 (*c_N_* = 0 and *b_N_* = 0, respectively). First, symbiotic evolution depends on the cost-to-benefit ratio in the following order: (i) “No evolution” (Figure 5, gray), (iii) “Intermediate evolution” (blue), (iv)–(vi) “Coexistence of cooperators and cheaters” (orange, purple, and green), and (ii) “Maximum evolution” (magenta). That is, the stronger the benefit (*b*) relative to the cost (*c*), the stronger the symbiotic relationship. Furthermore, *b_N_* inhibits the evolution of nitrogen-fixing bacteria; for example, the parameter region of case (ii) tends to decrease as *b_N_* increases. In contrast, *c_N_* promotes the emergence of cheating rhizobia, as shown by the expansion of cases (iv)–(vi) as *c_N_* becomes stronger.

### 3.4 Cost–Benefit Balance

**Figure 6:**
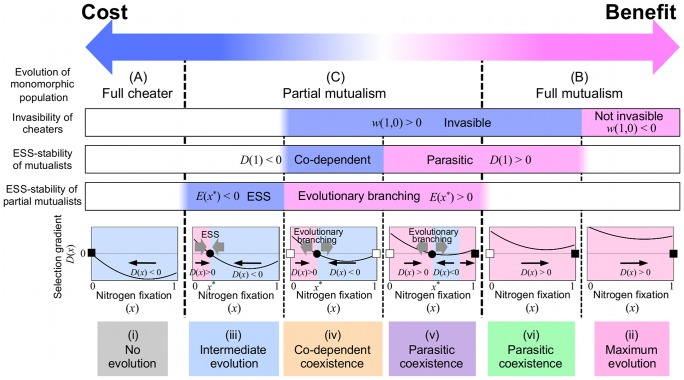
Cost–benefit balance in the symbiosis evolution. Cost–benefit balance determines the evolution of the symbiotic system by affecting various evolutionary features, such as selection gradient *D*(*x*), *w*(1,0): invasibility of cheaters in a population of mutualists, *E*(*x* ^*^): ESS-stability of partial mutualists, and *D*(1): ESS-stability of mutualists. Thereby, the evolutionary outcomes obtained in our model can be classified into six cases (i)–(vi) according to the cost–benefit balance. For details see text. The selection gradient determines the direction of evolution, such that a monomorphic population evolves towards larger strategies if *D*(*x*)>0 but towards smaller strategies if *D*(*x*)<0 (black arrows). Circles indicate singular strategies that are CS (i.e. *D*(*x* ^*^) = 0 and *D*′(*x* ^*^)<0). Filled squares correspond to cheaters (*x* = 0) or mutualists (*x* = 1) that are locally ESS-stable (i.e. *D*(0)<0 or *D*(1)>0, respectively), and open squares correspond to those that their strategy is not ESS-stable but can coexist with the other strategy.

As discussed above, the cost–benefit balance determines the evolutionary behavior of the symbiotic system. Here we summarize the evolutionary outcomes obtained in our model (Figure 6). According to the selection gradient *D*(*x*) (which defines the direction of evolution) (Figure 6, black arrows), an initially monomorphic population will evolve into three distinct nitrogen fixation activities: (A) full cheater with *x* = 0 if the cost is sufficiently strong (case (i)), (B) full mutualism with *x* = 1 if the cost is weakened (cases (ii)/(vi)), and (C) partial mutualism with *x* = *x* ^*^ at intermediate cost (cases (iii)–(v)). The evolution of these monomorphic populations is affected and classified by their various evolutionary features. The emergence of a fully mutualistic population depends on whether cheaters evolving by null mutation can invade the population (*w*(1,0): invasibility of cheaters). That is, a population of mutualists is stably maintained without invasion under weak cost conditions (*w*(1,0)<0; case (ii)). As the cost is strengthened, mutualists and cheaters can coexist because cheaters can successfully invade under this condition (*w*(1,0)>0; case (vi)).

However, the emergence of a partial mutualistic population depends on the occurrence of “evolutionary branching” (or sympatric speciation) (i.e. *E*(*x* ^*^): ESS-stability of partial mutualists; see Figure 6, gray arrows). A partially mutualistic population persists without invasion by mutants under relatively strong cost conditions (*E*(*x* ^*^)<0; case (iii)). As the cost is weakened, evolutionary branching creates a population of co-existent mutualists and cheaters (*E*(*x* ^*^)>0; cases (iv)/(v)). The cheaters in this type of coexistence can establish either a co-dependent or parasitic relationship with their mutualistic counterparts (i.e. *D*(1): ESS-stability of mutualists). In the absence of cheaters, a population of mutualists can stably exist under weaker cost (*D*(1)>0; case (v)) but cannot maintain nitrogen fixation activity under stronger cost (*D*(1)<0; case (iv)).

### 3.5 Efficiency of Nitrogen Fixation

#### 3.5.1 Assuming a linear cost function

**Figure 7:**
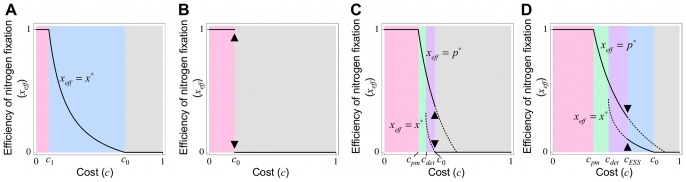
Efficiency of nitrogen fixation. The efficiency of nitrogen fixation (*x_eff_*) decreases with increasing cost *c*. **(A)** This decrease is continuous for a linear cost function (*c_N_* = 0). **(B–D)** If the benefit function is also linear (*b_N_* = 0), the decrease is discontinuous (arrowheads) at the transition between cases (i) and (ii) **(B)**, cases (i) and (v) **(C)**, and cases (iii) and (v) **(D)**. The parameter regions of cases (i), (ii), (iii), (v) and (vi) are indicated in gray, magenta, blue, purple, and green, respectively. Parameters are: *b* = 3.5 (A), 1.0 (B), 2.0 (C), and 4.0 (D); *b_N_* = 0.2 (A) and 0.0 (B–D); *c_N_* = 0.0 (A) and 0.5 (B–D); *n* = 5.

The efficiency of nitrogen fixation (*x_eff_*) relates to the average strategy adopted by the rhizobial population. Because a monomorphic population evolves in cases (i)–(iii), the efficiency in the linear cost function is described by
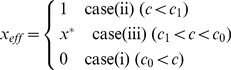
(8)where *x* ^*^ is described in Text S3.3. The efficiency continuously decreases as the cost *c* increases (Figure 7A).

#### 3.5.2 Assuming a linear benefit function

When full cooperators (*x* = 1) and full cheaters (*x* = 0) coexist in cases (iv)–(vi), the efficiency of nitrogen fixation in the linear benefit function depends on the proportion of cooperators, i.e.
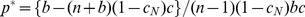
(9)(for details see Text S4.8). The efficiency then becomes
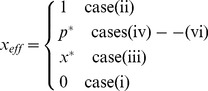
(10)where x^*^ is described in Text S4.4. As observed in the linear cost function, reducing the cost *c* increases the efficiency, but an interesting discontinuity occurs at the transition between case (i)/(iii) and case (ii)/(iv)/(v)/(vi) (Figure 7B–D).

### 3.6 Effect of Root Nodule Number

**Figure 8:**
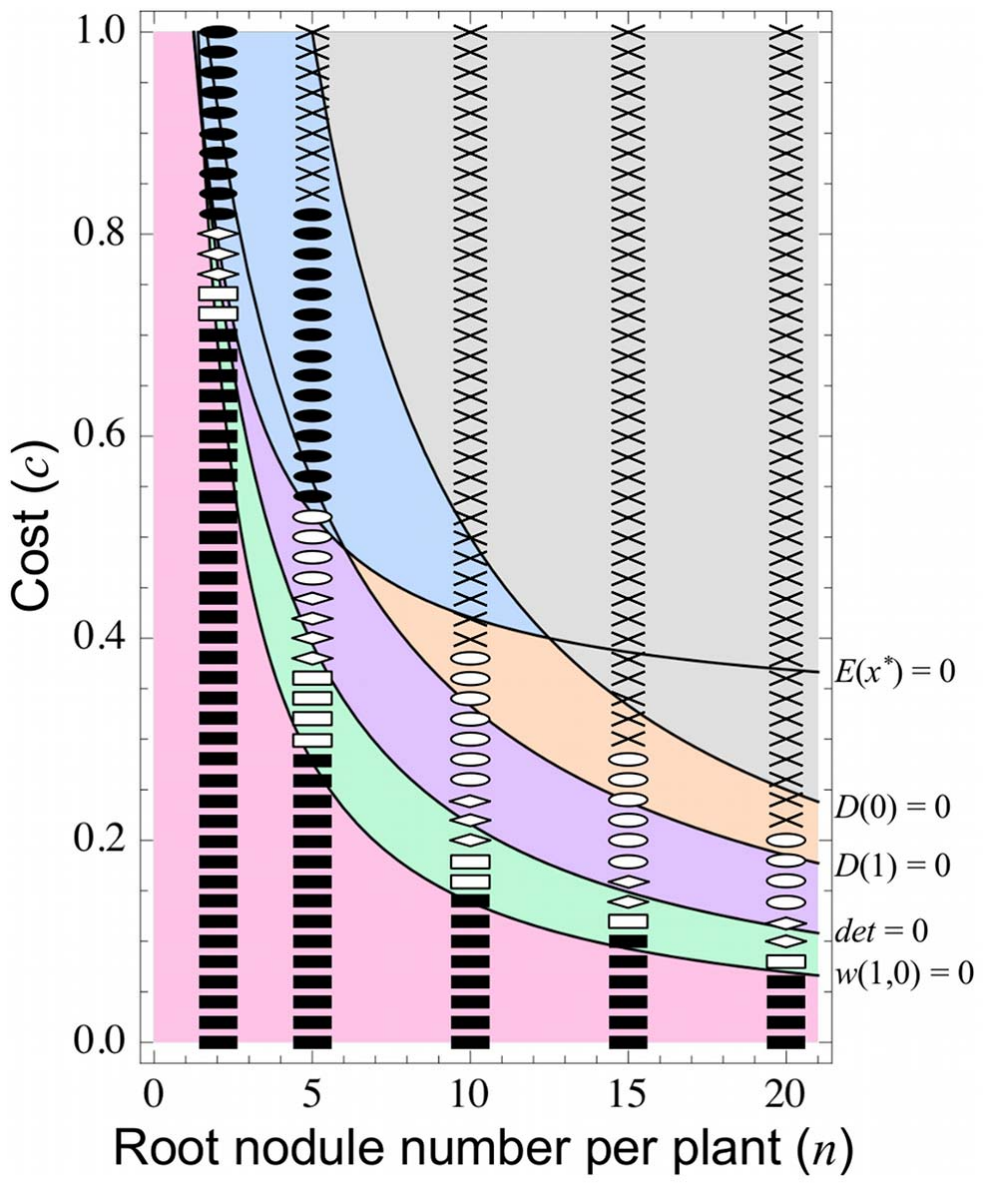
Effect of root nodule number. As the nodule number (*n*) on a host root increases, the symbiotic relationship evolves less easily. The parameter regions of case (ii) (magenta) and case (iii) (blue) decrease while that of case (i) (gray) increases. However, cheating rhizobia emerge more easily, as shown by the expanding parameter region in which cooperators and cheaters coexist (cases (iv)–(vi); orange, purple, and green). Theoretical and numerical predictions are indicated respectively by gray area and crosses (case (i)), magenta area and closed squares, (case (ii)), blue area and closed circles (case (iii)), orange area and open circles (case (iv)), purple area and diamonds (case (v)), and green area and open squares (case (vi)). Parameters are: *b* = 5.0, *b*  = 0.0, *c_N_* = 0.4.

Overcrowding of nodules on a host is expected to impede the mutualistic relationship, because the effect of an individual rhizobium on the benefit function *B*(*x*) weakens as the nodule number (*n*) increases (Eq. (1)). Supporting this prediction, the parameter conditions of case (i) expand with increasing *n* (Figure 8, gray) while those of cases (ii) and (iii) reduce (magenta and blue). Furthermore, higher nodule numbers promote the emergence of cheaters; the parameter area occupied by cases (iv)–(vi) is absent at *n* = 1 but expands as *n* increases (orange, purple, and green). These results suggest that, as nodule number increases, the symbiotic relationship destabilizes and is less easily established, while cheating rhizobia readily emerge.

### 3.7 Effect of Partner Choice

Partner choice is often described as the preferential selection of colonizers by the host plant [9]–[11]. To examine the effect of partner choice, we introduce a parameter *α*(*x*): the probability of accepting rhizobial colonization (where 0≤*α*(*x*)≤1 and *α*′(*x*)>0). We then consider the invasibility of rare mutants with nitrogen fixing ability *y* into the resident population with *x*. The mutant colonizes the host at a *α*(*y*)/*α*(*x*)-fold different rate than in the absence of partner choice (Figure 1B). Equivalently, partner choice affects the fitness of the mutant (*F_x_*(*y*)) by a factor of *α*(*y*)/*α*(*x*), so that
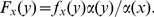
(11)


Accordingly, we obtain:
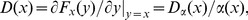
(12a)

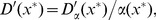
(12b)

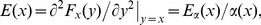
(12c)where 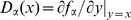, 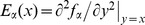, 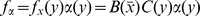, and *x* ^*^ is an evolutionarily singular strategy (i.e. *D*(*x* ^*^) = 0). This result indicates that a singular strategy (satisfying *D*(*x* ^*^) = 0) and its CS and ESS stabilities (which depend on the signs of *D*′(*x* ^*^) and *E*(*x* ^*^), respectively) can result from a modified fitness function 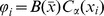, where 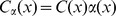 is a modified cost function. Therefore, similarly to host sanction, partner choice promotes symbiotic evolution by influencing the cost function.

### 3.8 Effect of Mixed Nodule Populations

Mixed nodules colonized by multiple symbionts are common in the laboratory [40]–[42] and are also observed under field conditions [43]. Mixed nodule populations are thought to reduce the evolutionary effects of host sanction and thereby promote the emergence of ineffective rhizobia [10], [13], [16], [34]. To examine the effects of mixed nodule populations in our model, we introduce a parameter *β* (0≤*β*≤1), specifying the frequency of mixed nodules (or doubly colonized nodules). Each nodule is colonized by two rhizobial strains with probability *β*; otherwise it is colonized by a single strain (with probability 1–*β*).

In a mixed nodule, two strains adopting strategies *x*_1_ and *x*_2_ would proliferate at rates depending on their relative fitness *C*(*x_i_*) (*i* = 1 or 2). Thus, we assume their proportion in the population as 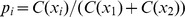. Accordingly, the nitrogen fixation activity of the mixed nodule, which contributes to the average nitrogen fixation (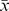) in the benefit function, is given by 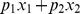. Furthermore, the fitness of each strain depends on its proportion (*p_i_*) in the mixed nodule and is simply defined as 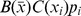, which is reduced to single colonization (i.e. 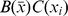) and 0 when *p_i_* = 1 and *p_i_* = 0, respectively.

Now consider that mutants with nitrogen fixation ability *y* invade a resident population with ability *x*. If the mutant is rare, the probability that the host plant is colonized by multiple mutants is very low and can be neglected. Thus, root nodules will harbor single *y* colonies and colonies containing *x* and *y* with relative frequencies of (1–*β*) and 2*β*, respectively (Figure 1C). The fitness of the mutant in a single colonization is 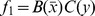 where 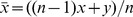, and that in a double colonization is 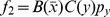, where 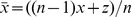, 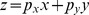, 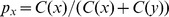, and 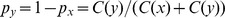. Consequently, the expected fitness of mutant *y* is
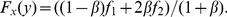
(13)


Thus we obtain the selection gradient
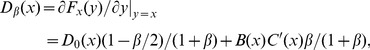
(14)where 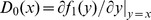 is the gradient in a pure population (i.e. *β* = 0). The second term on the right-hand side of Eq. (14) is always negative because *C*′(*x*)<0, implying that mixed nodule populations inhibit the evolution of nitrogen fixation.

**Figure 9:**
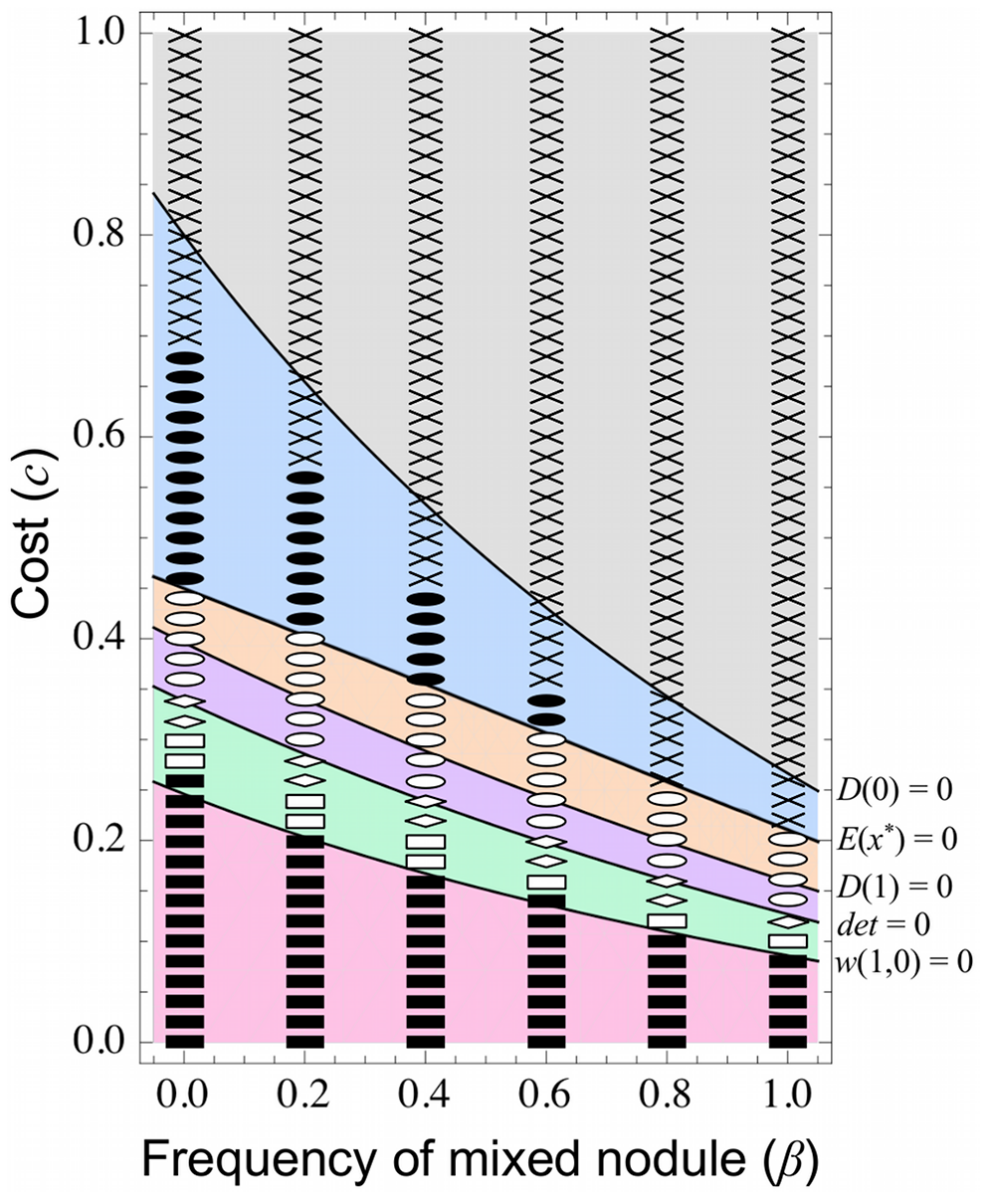
Effect of mixed nodule populations. Our model predicts that mixed nodule populations destabilize the symbiotic relationship (see section 3.8 for details). This prediction is supported by numerical simulations. Theoretical and numerical predictions are indicated respectively by gray area and crosses (case (i)), magenta area and closed squares, (case (ii)), blue area and closed circles (case (iii)), orange area and open circles (case (iv)), purple area and diamonds (case (v)), and green area and open squares (case (vi)). Parameters are: *b* = 4.0, *b_N_* = 0.0, *c_N_* = 0.35, *n* = 5.

This prediction is supported by numerical simulations, in which the parameter region of “No evolution” expands as *β* increases, while that of “Maximum evolution” decreases (Figure 9, gray and magenta). In contrast, mixed nodule populations exert no positive effects on mutualistic–cheater co-existence, because the parameter range of cases (iv)–(vi) is not largely influenced by *β* (Figure 9, orange, purple, and green). However, as *β* increases, this parameter region moves toward lower cost (*c*). In this scenario, cheating rhizobia will either emerge or disappear in mixed nodule populations, depending on the parameter conditions. For example, setting *c* = 0.2 in Figure 9, cheaters cannot exist in mixed nodule populations that establish with low probability (*β*<*β*_1_≈0.217; case (ii), magenta), but can emerge when the probability of mixed populations is higher (*β*>*β*_1_; cases (iv)–(vi), green, purple, and orange). Conversely, when *c* = 0.4, cheaters can coexist with mutualists at lower *β* (<*β*_2_≈0.212; cases (iv) and (v), orange and purple), but are excluded at higher *β* (>*β*_2_; cases (i) and (iii), blue and gray).

## Discussion

In symbiotic relationships, the participating organisms provide mutual benefits to each other. Such positive fitness feedback reinforces their mutualistic interaction. However, symbiotic systems encourage the emergence of selfish parasitic cheaters, whose performance undermines the system. Thus, symbiotic systems are simultaneously exposed to promoting and destabilizing forces, analogous to benefit and cost in game theory.

**Figure 10:**
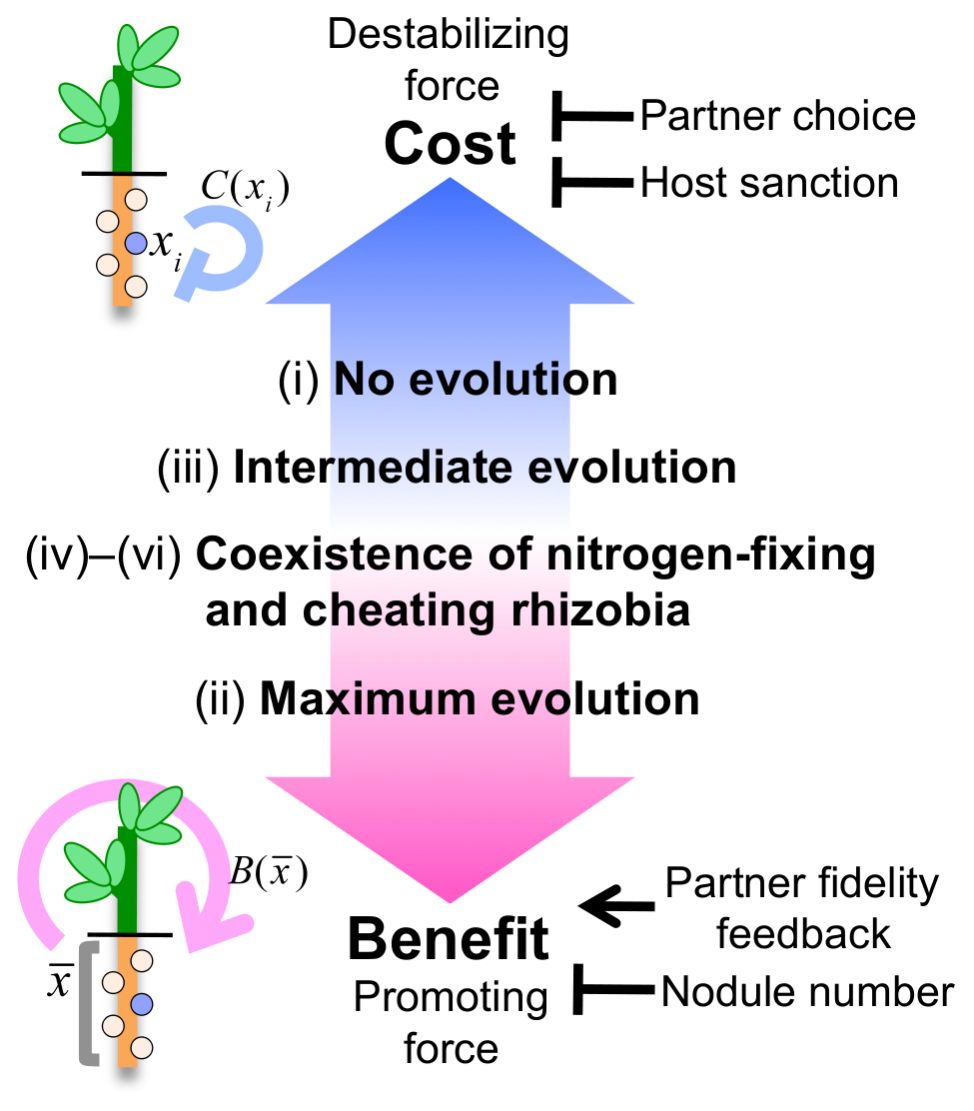
Model for the evolution of the legume–rhizobia symbiosis. The evolution of the legume–rhizobia symbiosis depends on the cost–benefit balance. As the benefit strengthens relative to the cost, the evolutionary outcome shifts in the following order: (i) “No evolution”, (iii) “Intermediate evolution”, (iv)–(vi) “Coexistence of nitrogen-fixing and cheating rhizobia”, and (ii) “Maximum evolution”. The symbiotic relationship is reinforced by partner fidelity feedback, which strengths the benefit, and by host sanction and partner choice, which diminish the cost. In addition, as the number of nodules on a root increases, symbiotic rhizobia are displaced by selfish cheaters.

One of the most famous symbioses occurs between legumes and rhizobia. Here, we intensively investigated how benefit and cost influence the evolution of this symbiosis, and the conditions required for establishing the symbiotic relationship. According to our model, stable mutualism depends on the cost–benefit balance (Figures 6 and 10). That is, a tight symbiotic relationship emerges when the beneficial effect is much stronger than the cost (case (ii)), but is dissolved under the opposite condition of relatively strong cost (case (i)). In the intermediate condition, where benefit is approximately offset by cost, more complicated behaviors emerge such as imperfect symbiotic interactions (case (iii)) and the coexistence of cooperators and cheaters (cases (iv)–(vi)). As the benefit strengthens relative to the cost, the evolutionary outcome shifts in the order of “No evolution”, “Intermediate evolution”, “Coexistence of cooperators and cheaters”, and “Maximum evolution” (Figure 10).

Ineffective rhizobia with little or no nitrogen fixation activity are widely distributed in natural environments [5]–[8], and their emergence and persistence has aroused much discussion [4], [10], [44]–[46]. Our model showed that cheaters can emerge when the benefit and cost are balanced (Figure 10, cases (iv)–(vi)). Under such conditions, nitrogen-fixing cooperators can co-exist with cheaters and maintain stable symbiotic relationships. Therefore, the emergence of cheating bacteria does not necessarily disrupt a symbiotic relationship.

According to our model, cheating rhizobia can be classified into “co-dependent” or “parasitic”, which exert different effects on the system. The presence of “co-dependent” cheaters stabilizes a symbiotic system (Figure 4E), while “parasitic” cheaters produce no effect on the system (Figure 4F, G). However, in natural environments, these classes of cheaters may be difficult to distinguish. One reason is that cheaters are spontaneously generated by mutations; thus, the mutualistic effects of cheaters in the complete absence of the null mutation are not easily examinable. In addition, cheaters could easily transfer between “co-dependent” and “parasitic” under small changes of parameter conditions, because the classes are adjacent in the parameter space (Figures 4A, 8, 9, and S2). Because parameter values can be perturbed by environmental fluctuations, cheating rhizobia may continuously alternate between “co-dependent” and “parasitic”.

As mentioned above, the persistence of the legume–rhizobia symbiosis in nature, despite the ubiquity of ineffective rhizobia, has aroused much interest. Various mechanisms that stabilize the symbiotic relationship have been proposed; in particular, partner fidelity feedback [1]–[4], partner choice [4], [9]–[12], and host sanction [4], [10], [13]–[16]. In our model, partner fidelity feedback provides a benefit (or promoting force), while host sanction and partner choice reduce the cost function *C*(*x*) (or destabilizing force). Therefore, our model suggests that these factors strengthen the symbiotic interaction in opposite ways; partner fidelity feedback reinforces the benefit while host sanction and partner choice ameliorate the cost (Figure 10). Thus, although the legume–rhizobia symbiosis may be maintained by a single mechanism, it is rather more likely to be cooperatively reinforced by various stabilizing mechanisms.

Mixed nodule populations are thought to reduce the evolutionary effects of host sanction, and thereby encourage the persistence of cheating rhizobia [10], [13], [16], [34]. However, our model predicts that mixed nodule populations exert destabilizing effects on the symbiotic relationship, and can cause cheaters to either emerge or vanish depending on the parameter conditions. This evidence suggests that mixed nodule populations produce both positive and negative effects on the emergence of cheating rhizobia in different situations.

In this paper, we assumed a decreasing cost function (i.e. *C*′(*x*)<0), implying that more cheating rhizobia proliferate more efficiently if they infect the same host plant, because the fitness is given by 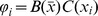 (Eq. (1)). Interestingly, under these conditions, ineffective rhizobia do not necessarily evolve to persistence, and tight mutualistic interaction is not prohibited (for example case (ii)). Thus, while cheaters are benefitted by a growth advantage, they do not necessarily co-evolve with cooperators or cause collapse of the symbiotic system. This result reconfirms that the cost–benefit balance, rather than the cost alone, is important for symbiotic evolution (Figure 10).

However, double inoculation experiments have shown that nitrogen-fixing strains proliferate with equal or superior efficacy to ineffective strains colonizing the same plant seedling [4], [11], [15], [33], [47]–[50]. This experimental observation may be attributed to various synergistic effects, but ultimately suggests that the cost function is not a simple decreasing function of nitrogen fixation. Under such conditions (i.e. *C*′(*x*)≥0), our theory predicts that rhizobial populations always evolve to the maximum activity of nitrogen fixation (see Text S2). However, this prediction contradicts the ecological fact that ineffective rhizobia are widespread in natural environments [5]–[8]. Possibly, the benefit and cost functions are not simple increasing or decreasing function of *x* as we have assumed, but respond with more complexity to nitrogen resources. Furthermore, our highly-simplified model ignores a number of effects such as plant strategy dynamics, symbiont growth in the soil, spatial heterogeneity, and environmental fluctuations. Incorporation of these effects into our model might introduce greater complexity to the evolution of legume–rhizobia symbiosis.

In our model, rhizobia fitness is influenced by both the average nitrogen fixation of the rhizobia infecting a host plant (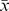) and individual rhizobium activity (*x_i_*) (Eq. (1)), here denoted by “systemic” and “local” effects, respectively. Both effects are essential for coexistence of nitrogen-fixing and cheating rhizobia, because coexistence cannot emerge without their mutual cooperation (for details see Text S5). Although the benefit and cost functions in this paper are simply given by the systemic and local effects, respectively, they could take more complex general forms incorporating both effects; for instance, 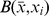 and 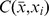. If the systemic and local effects in these functions are approximately separable from each other (i.e. 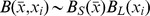 and 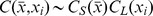), the fitness can be written as
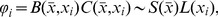
(15)where 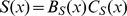 and 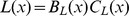 are functions of the systemic and local effects, respectively. Replacing *S*(*x*) and *L*(*x*) respectively with *B*(*x*) and *C*(*x*), Eq. (15) takes the fitness form of Eq. (1); hence, the evolutionary dynamics described by Eq. (15) can be understood within the framework described in this paper. For example, the benefit (or partner fidelity feedback) might include both local and systemic effects. The local beneficial effect (i.e. *B_L_*(*x_i_*)) would eventually reinforce the symbiotic relationship by affecting the local function *L*(*x*) of Eq. (15), similarly to the weakening of the cost function *C*(*x*) by host sanction and partner choice in Eq. (1).

In this paper, we constructed and investigated the evolutionary dynamics of the legume–rhizobia symbiotic system, driven by the opposite effects of promoting force (or benefit) and destabilizing force (or cost). We then determined a comprehensive set of conditions under which the symbiotic relationship is established and cheating bacteria emerge. Our findings provide a theoretical basis for understanding how the legume/rhizobium symbiosis evolves. However, as mentioned above, our model is extremely simplified, and evolutionary behaviors in the natural environment are expected to be much more complicated than those seen in our model. By incorporating such effects into our model, we could better understand the evolution of symbiotic systems.

## Supporting Information

Figure S1 **Effect of** ***b_N_*** **, assuming a linear cost function.** If a linear cost function (*c_N_* = 0) is assumed, nonlinearity in the benefit function (*b_N_*) hinders symbiotic evolution. As *b_N_* increases, the parameter region of case (ii) “Maximum evolution” (magenta) decreases while that of case (iii) “Intermediate evolution” (blue) increases. This prediction is consistent with numerical simulations; cases (i), (ii) and (iii) are indicated by crosses, squares, and circles, respectively. Parameters are: *b* = 5.0, *c_N_* = 0.0, *n* = 5.
(TIF)

Figure S2 **Effect of** ***c_N_*** **, assuming a linear benefit function.** If a linear benefit function (*b_N_* = 0) is assumed, nonlinearity in the cost function (*c_N_*) promotes symbiosis evolution. As *c_N_* increases, the parameter region of case (ii) “Maximum evolution” (magenta) increases, while that of case (iii) “Intermediate evolution” (blue) decreases. In addition, *c_N_* promotes the emergence of cheaters. The parameter region in which nitrogen-fixing and cheating rhizobia coexist (cases (iv)–(vi); orange, purple, and green areas, respectively) is absent when *c_N_*<*c_N_* ^*^ = *b*/(*n*+(*n*+2) *b*), but otherwise increases as *c_N_* increases. These predictions are consistent with numerical simulations; cases (i), (ii), (iii), (iv), (v) and (vi) are indicated by crosses, closed squares, closed circles, open circles, diamonds, and open squares, respectively. Parameters are: *b* = 5.0, *b_N_* = 0.0, *n* = 5.
(TIF)

Text S1 **Theoretical framework of adaptive dynamics.**
(PDF)

Text S2 **Increasing cost function (** ***C′*** **(** ***x*** **)>0).**
(PDF)

Text S3 **Linear cost function (** ***c_N_*** **= 0).**
(PDF)

Text S4 **Linear benefit function (** ***b_N_*** **= 0).**
(PDF)

Text S5 **Systemic and local effects of nitrogen fixation.**
(PDF)
