# Supplementary figures and images for: Evolutionary Dynamics of Nitrogen Fixation in the Legume–Rhizobia Symbiosis
Source: PLoS One. 2014 Apr 1;9(4):e93670. doi: 10.1371/journal.pone.0093670 (PMC3972148; doi:10.1371/journal.pone.0093670)

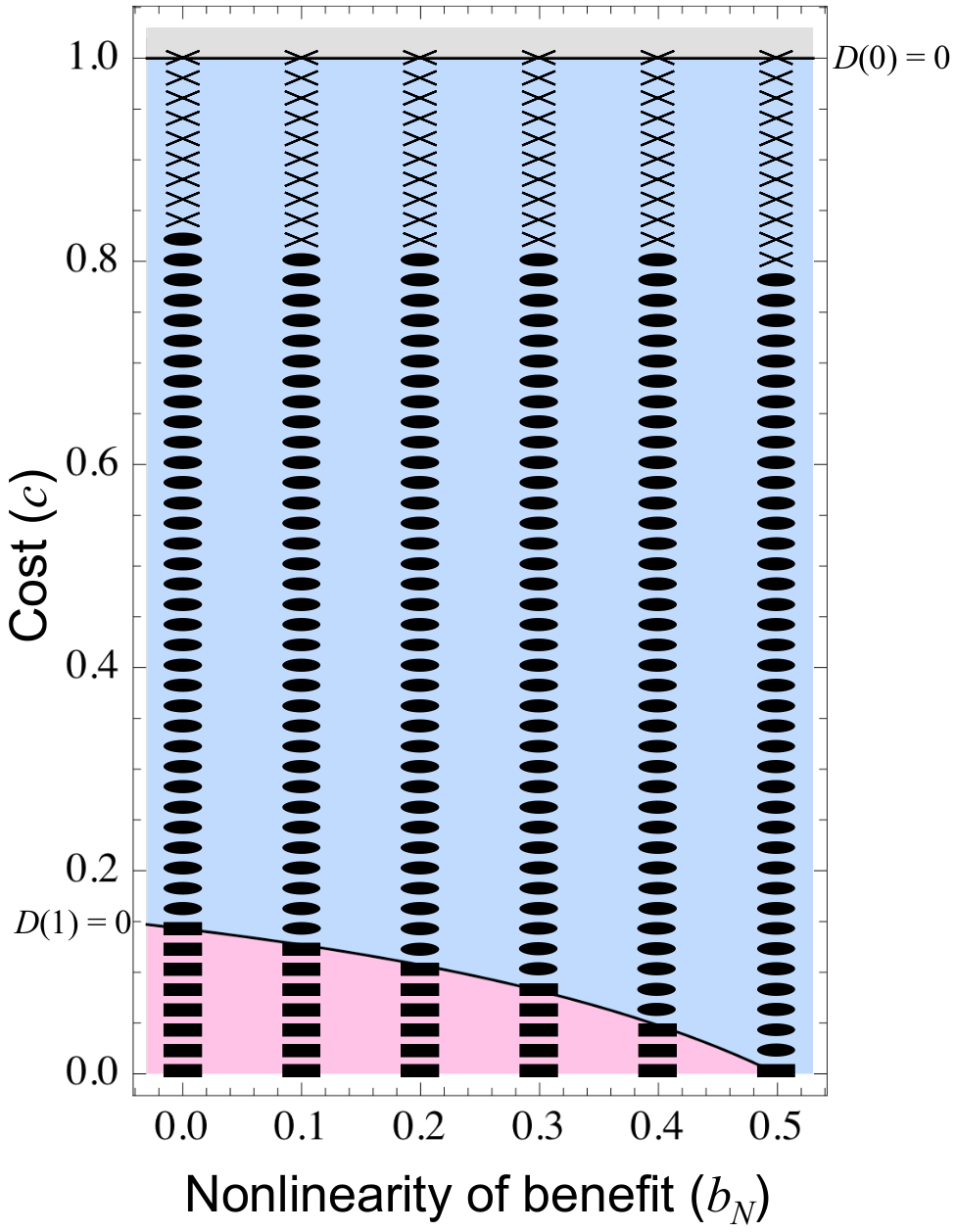

Supplement: Figure S1 — Effect of bN , assuming a linear cost function. If a linear cost function (cN = 0) is assumed, nonlinearity in the benefit function (bN) hinders symbiotic evolution. As bN increases, the parameter region of case (ii) “Maximum evolution” (magenta) decreases while that of case (iii) “Intermediate evolution” (blue) increases. This prediction is consistent with numerical simulations; cases (i), (ii) and (iii) are indicated by crosses, squares, and circles, respectively. Parameters are: b = 5.0, cN = 0.0, n = 5. (TIF) [file pone.0093670.s001.tif]

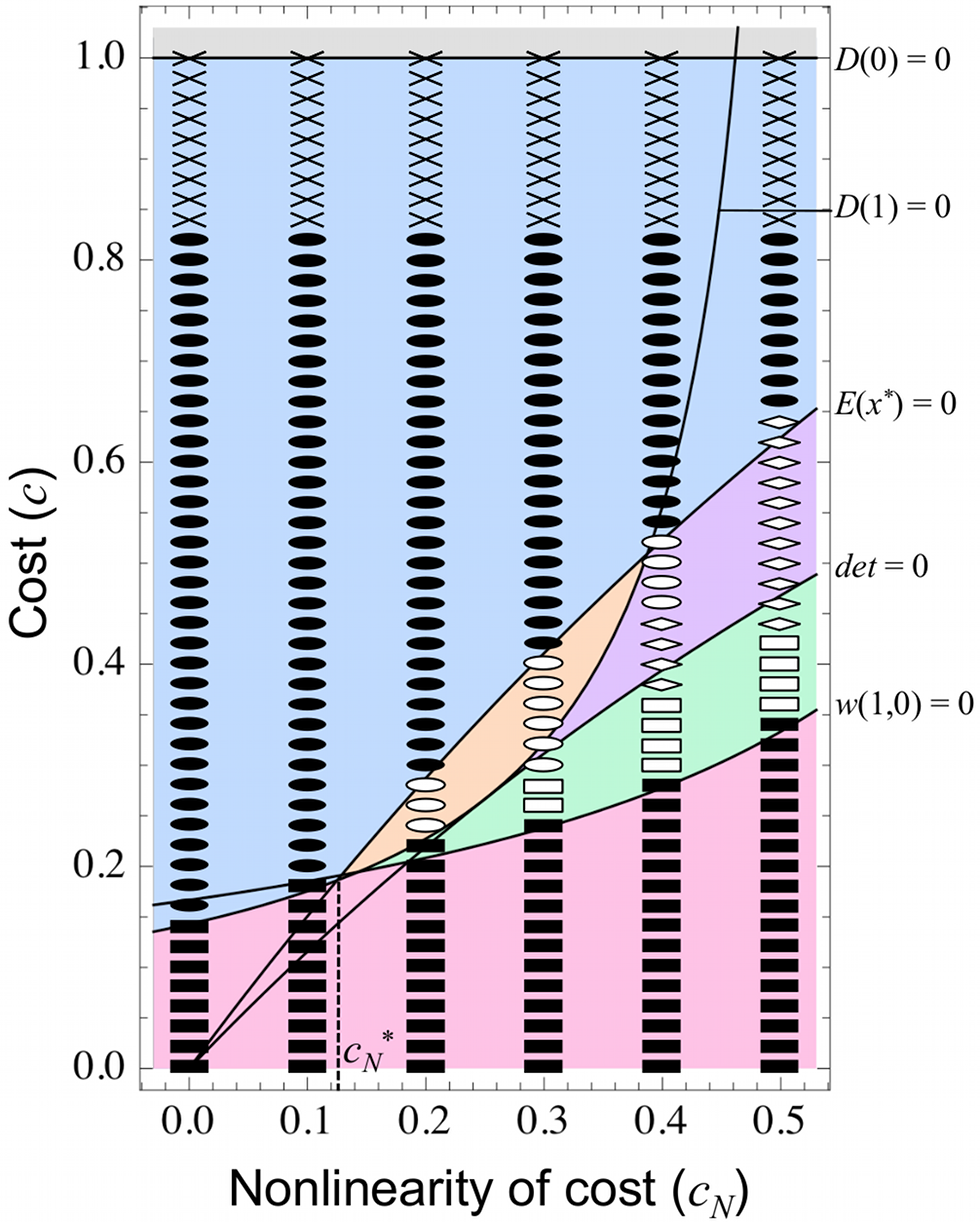

Supplement: Figure S2 — Effect of cN , assuming a linear benefit function. If a linear benefit function (bN = 0) is assumed, nonlinearity in the cost function (cN) promotes symbiosis evolution. As cN increases, the parameter region of case (ii) “Maximum evolution” (magenta) increases, while that of case (iii) “Intermediate evolution” (blue) decreases. In addition, cN promotes the emergence of cheaters. The parameter region in which nitrogen-fixing and cheating rhizobia coexist (cases (iv)–(vi); orange, purple, and green areas, respectively) is absent when cN<cN * = b/(n+(n+2) b), but otherwise increases as cN increases. These predictions are consistent with numerical simulations; cases (i), (ii), (iii), (iv), (v) and (vi) are indicated by crosses, closed squares, closed circles, open circles, diamonds, and open squares, respectively. Parameters are: b = 5.0, bN = 0.0, n = 5. (TIF) [file pone.0093670.s002.tif]
