# Supplementary material for: Evolutionary Dynamics of Nitrogen Fixation in the Legume–Rhizobia Symbiosis
Source: PLoS One. 2014 Apr 1;9(4):e93670. doi: 10.1371/journal.pone.0093670 (PMC3972148; doi:10.1371/journal.pone.0093670)
Supplement: Text S1 — Theoretical framework of adaptive dynamics. (PDF) [file pone.0093670.s003.pdf]

Evolutionary Dynamics of Nitrogen Fixation in the Legume–Rhizobia Symbiosis

Hironori Fujita[1*], Seishiro Aoki[2], Masayoshi Kawaguchi[1,3]

[1] Division of Symbiotic Systems, National Institute for Basic Biology, National Institute for Natural Sciences, Okazaki 444-8585, Japan

[2] Department of General Systems Studies, Graduate School of Arts and Sciences, University of Tokyo, 3-8-1 Komaba, Meguro-ku, Tokyo 153-8902, Japan

[3] Department of Basic Biology, School of Life Science, Graduate University for Advanced Studies (SOKENDAI), Okazaki 444-8585, Japan

[*] E-mail: hfujita@nibb.ac.jp

Supporting Information

Text S1. Theoretical framework of adaptive dynamics

S1.1. Invasibility of mutants

Here, we briefly introduce the theoretical framework of adaptive dynamics. This framework underlies the evolutionary dynamics of a quantitative trait with a frequency-dependent selection [1,2]. Let $f_x(y)$ be the growth rate (or fitness) of rare mutants adopting strategy y, when they invade a resident population adopting strategy x. The relative growth rate between the mutant and the resident is given by

$$w(x,y) \equiv f_x(y) - f_x(x)\,. \tag{S1.1}$$

This function determines the invasibility of the mutant into the resident population. If $w(x,y) > 0$, the mutant can invade; if $w(x,y) < 0$, it cannot.

S1.2. Selection gradient

If x and y are very similar to one another (i.e. $|y - x| << 1$), the relative growth rate is

expressed by

$$w(x,y) = D(x)(y-x) + E(x)(y-x)^2/2 + O(y-x)^3, \qquad \text{(S1.2)}$$

where $D(x) \equiv \partial f_x(y)/\partial y\big|_{y=x}$ and $E(x) \equiv \partial^2 f_x(y)/\partial y^2\big|_{y=x}$. Thus, the invisibility is approximately linear, and we have $w(x,y) = (y-x)D(x)$. Here, $D(x)$ is termed the "selection gradient" because its sign represents the direction of evolution. If $D(x) > 0$, the resident population with x can be invaded by mutants with $y > x$ because $w(x,y) > 0$; conversely, if $y < x$, the mutants cannot invade because $w(x,y) < 0$. In this way, the population evolves towards larger strategies by repeat replacement of existing residents by higher-performing mutants. Conversely, if $D(x) < 0$, only mutants with $y < x$ can invade and replace the resident population, which drives the population toward smaller strategies.

S1.3. Evolutionarily singular strategy

The strategy x^* satisfying $D(x^*) = 0$, where mutant invasibility is reversed, is called an evolutionarily singular strategy. If a singular strategy x^* satisfies $D'(x^*) < 0$ (i.e. $D(x) > 0$ for $x < x^*$ and $D(x) < 0$ for $x > x^*$), then x^* is "convergence stable" (CS). In this case, a monomorphic population with a similar phenotype can be invaded by mutants whose strategies approach x^*. Conversely, if $D'(x^*) > 0$ then x^* is convergence unstable and the population can be invaded by mutants adopting quite different strategies from x^*.

S1.4. Evolutionarily stable strategy (ESS)

If a singular strategy x^* is CS (i.e. $D(x^*) = 0$ and $D'(x^*) < 0$), the evolutionary stability of the population with x^* depends on the sign of $w(x^*, y) = E(x^*)(y-x^*)^2/2$, namely,

the sign of $E(x^*)$. If x^* satisfies $E(x^*) < 0$ (i.e. $w(x^*,y) < 0$) for any nearby y, then x^* is called an “evolutionarily stable strategy” (ESS). In this case, the population is robust against invasion by mutants with similar strategies. Accordingly, when the singular strategy x^* is CS and ESS (i.e. $D(x^*) = 0$, $D'(x^*) < 0$, and $E(x^*) < 0$), a monomorphic population evolves toward x^*, where it is stably maintained without invasion by similar-phenotype mutants.

S1.5. Evolutionary branching

Conversely, if $E(x^*) > 0$ (i.e. $w(x^*,y) > 0$ for any nearby y), then x^* is ESS-unstable. Interestingly, such a singular strategy, that is CS but not ESS-stable (i.e. $D(x^*) = 0$, $D'(x^*) < 0$, and $E(x^*) > 0$), can induce species speciation or “evolutionary branching”, in which a monomorphic population with x^* is invaded by nearby mutants and subsequently forms two subpopulations with higher and lower strategies than the original x^*.

References

1. Geritz S, Kisdi E, Meszena G, Metz J (1998) Evolutionarily singular strategies and the adaptive growth and branching of the evolutionary tree. Evol Ecol 12: 35-57.
2. Waxman D, Gavrilets S (2005) 20 questions on adaptive dynamics. J Evol Biol 18: 1139-1154.
