# Supplementary material for: Evolutionary Dynamics of Nitrogen Fixation in the Legume–Rhizobia Symbiosis
Source: PLoS One. 2014 Apr 1;9(4):e93670. doi: 10.1371/journal.pone.0093670 (PMC3972148; doi:10.1371/journal.pone.0093670)
Supplement: Text S2 — Increasing cost function ( C′ ( x )>0). (PDF) [file pone.0093670.s004.pdf]

Evolutionary Dynamics of Nitrogen Fixation in the Legume–Rhizobia Symbiosis

Hironori Fujita[1*], Seishiro Aoki[2], Masayoshi Kawaguchi[1,3]

[1] Division of Symbiotic Systems, National Institute for Basic Biology, National Institute for Natural Sciences, Okazaki 444-8585, Japan

[2] Department of General Systems Studies, Graduate School of Arts and Sciences, University of Tokyo, 3-8-1 Komaba, Meguro-ku, Tokyo 153-8902, Japan

[3] Department of Basic Biology, School of Life Science, Graduate University for Advanced Studies (SOKENDAI), Okazaki 444-8585, Japan

[*] E-mail: hfujita@nibb.ac.jp

Supporting Information

Text S2. Increasing cost function ($C'(x) > 0$)

Because the benefit function $B(x) \geq 0$ is an increasing function of x, we have

$$B(\bar{x}) < B(x) \text{ for } y < \bar{x} < x \text{ and } B(\bar{x}) > B(x) \text{ for } y > \bar{x} > x, \tag{S2.1}$$

where $\bar{x} = ((n-1)x + y)/n$. If the cost function $C(x) \geq 0$ is a similarly increasing function of x, then

$$C(y) < C(x) \text{ for } y < x \text{ and } C(y) > C(x) \text{ for } y > x. \tag{S2.2}$$

From Eqs. (S2.1) and (S2.2), the following conditions must be met:

$$B(\bar{x})C(y) < B(x)C(x) \text{ for } y < x \text{ and } B(\bar{x})C(y) > B(x)C(x) \text{ for } y > x. \tag{S2.3}$$

Because the invasibility of mutant y is $w(x,y) = B(\bar{x})C(y) - B(x)C(x)$, we obtain

$$w(x,y) < 0 \text{ for } y < x \text{ and } w(x,y) > 0 \text{ for } y > x. \tag{S2.4}$$

This invasibility condition is identical to that of case (ii) “Maximum evolution” in section 3.1 (Figure 2C). That is, a resident population can be invaded and replaced by

mutants adopting higher strategies, but not by those adopting lower strategies. This result shows that the maximum activity of nitrogen fixation ($x = 1$) will evolve and stably persist if both $B(x)$ and $C(x)$ are increasing functions of x.
