# Supplementary material for: Evolutionary Dynamics of Nitrogen Fixation in the Legume–Rhizobia Symbiosis
Source: PLoS One. 2014 Apr 1;9(4):e93670. doi: 10.1371/journal.pone.0093670 (PMC3972148; doi:10.1371/journal.pone.0093670)
Supplement: Text S3 — Linear cost function ( cN = 0). (PDF) [file pone.0093670.s005.pdf]

Evolutionary Dynamics of Nitrogen Fixation in the Legume–Rhizobia Symbiosis

Hironori Fujita[1*], Seishiro Aoki[2], Masayoshi Kawaguchi[1,3]

[1] Division of Symbiotic Systems, National Institute for Basic Biology, National Institute for Natural Sciences, Okazaki 444-8585, Japan

[2] Department of General Systems Studies, Graduate School of Arts and Sciences, University of Tokyo, 3-8-1 Komaba, Meguro-ku, Tokyo 153-8902, Japan

[3] Department of Basic Biology, School of Life Science, Graduate University for Advanced Studies (SOKENDAI), Okazaki 444-8585, Japan

[*] E-mail: hfujita@nibb.ac.jp

Supporting Information

Text S3. Linear cost function (c_N = 0)

If the cost function is linear (i.e. $c_N = 0$), the selection gradient is

$$D(x) = \left\{(n+2)b_N bcx^2 - (c + nc + 2b_N)bx + b - nc\right\}/n\,, \tag{S3.1}$$

which is always a decreasing function of x because

$$D'(x) = -b\left\{(n+1)c(1-2b_N x) + 2b_N(1-cx)\right\}/n < 0\,. \tag{S3.2}$$

Furthermore, we have

$$E(x) = \partial^2 f_x(y)/\partial y^2\Big|_{y=x} = -2b\left\{nc(1-2b_N x) + b_N(1-cx)\right\}/n^2 < 0\,. \tag{S3.3}$$

Thus our model predicts the following three evolutionary behaviors.

S3.1. Case (i) No evolution

Because $D(x)$ is a decreasing function, we have $D(x) < 0$ for $0 < x < 1$ and $D(0) < 0$ (i.e. $c > c_0 = b/n$) (Figures 2A and S1, gray). This condition corresponds to case (i) "No

evolution” in section 3.1.1.

S3.2. Case (ii) Maximum evolution

The opposite case is $D(x) > 0$ for $0 < x < 1$, and $D(1) > 0$ (i.e. $c < c_1 = (1-2b_N)b \big/ \left\{n+(n+1-nb_N-2b_N)b\right\}$) (Figures 2A and S1, magenta). This condition corresponds to case (ii) “Maximum evolution” in section 3.1.2.

S3.3. Case (iii) Intermediate evolution

Between these two extremes (i.e. $c_1 < c < c_0$) (Figures 2A and S1, blue), a unique singular strategy $0 < x^* < 1$ is found:

$$x^* = (b-nc)\big/(n+1)bc \quad \text{(for } b_N = 0\text{)}, \tag{S3.4a}$$

$$x^* = \left\{b(c+nc+2b_N)-\sqrt{det}\right\}\big/2(n+2)b_N bc \quad \text{(for } b_N \neq 0\text{)}, \tag{S3.4b}$$

where $det = b^2(c+nc+2b_N)^2 - 4(n+2)b_N bc(b-nc)$. This singular strategy x^* is always CS and ESS-stable (i.e. $D'(x^*) < 0$ and $E(x^*) < 0$) (Eqs. (S3.2) and (S3.3)). This condition corresponds to case (iii) “Intermediate evolution” in section 3.1.3.

S3.4. Invasibility of cheating rhizobia

In the resident population with strategy x, the invasibility of cheating bacteria ($y = 0$) is determined by the sign of

$$w(x,0) = -xD(x) + x^2E(x)\big/2 - b_N bcx^3\big/n^2 . \tag{S3.5}$$

By Eq. (S3.3), the second term of the right hand side of Eq. (S3.5) is negative. Thus we have $w(1,0) < 0$ because $D(1) > 0$ in case (ii), and $w(x^*,0) < 0$ because $D(x^*) = 0$ in case (iii). This result shows that, in both cases (ii) and (iii), cheating rhizobia cannot invade the resident population.
