# Supplementary material for: Evolutionary Dynamics of Nitrogen Fixation in the Legume–Rhizobia Symbiosis
Source: PLoS One. 2014 Apr 1;9(4):e93670. doi: 10.1371/journal.pone.0093670 (PMC3972148; doi:10.1371/journal.pone.0093670)
Supplement: Text S4 — Linear benefit function ( bN = 0). (PDF) [file pone.0093670.s006.pdf]

Evolutionary Dynamics of Nitrogen Fixation in the Legume–Rhizobia Symbiosis

Hironori Fujita[1*], Seishiro Aoki[2], Masayoshi Kawaguchi[1,3]

[1] Division of Symbiotic Systems, National Institute for Basic Biology, National Institute for Natural Sciences, Okazaki 444-8585, Japan

[2] Department of General Systems Studies, Graduate School of Arts and Sciences, University of Tokyo, 3-8-1 Komaba, Meguro-ku, Tokyo 153-8902, Japan

[3] Department of Basic Biology, School of Life Science, Graduate University for Advanced Studies (SOKENDAI), Okazaki 444-8585, Japan

[*] E-mail: hfujita@nibb.ac.jp

Supporting Information

Text S4. Linear benefit function (b_N = 0)

If the benefit function is linear (i.e. $b_N = 0$), the selection gradient is

$$D(x) = \left\{ b - nc - c(b + nb - 2nc_N)x + (2n+1)c_N bcx^2 \right\} / n. \qquad \text{(S4.1)}$$

S4.1. Case (i) No evolution

If $D(0) < 0$ (i.e. $c > c_0 = b/n$), an initial population with $x = 0$ cannot be invaded by mutants adopting similar strategies and is therefore stably maintained (Figures 4A and S2, gray). Thus, nitrogen fixation activity does not evolve, and the population remains in the “No evolution” state (i.e. $D(0) < 0$) in section 3.2.

S4.2. Case (ii) Maximum evolution

Conversely, if $D(x) > 0$ for $0 \le x \le 1$, maximum nitrogen fixation activity ($x = 1$) evolves in the absence of null mutation. This condition is summarized as follows:

$D(0) > 0$ (i.e. $c < c_0$) if $axis < 0$ (i.e. $b < b_0$), (S4.2a)

$D(1) > 0$ (i.e. $c < c_1$) if $axis > 1$ (i.e. $b > b_1$), (S4.2b)

$det < 0$ (i.e. $c < c_{\text{det}}$) if $0 < axis < 1$ (i.e. $b_0 < b < b_1$), (S4.2c)

where $det = (b + nb - 2nc_N)^2 c^2 - 4(2n+1)(b - nc)c_N bc$ and $axis = (b + nb - 2nc_N)/2(2n+1)c_N b$ are the determinant and axis, respectively, of the quadratic function $nD(x)$, and $b_0 = 2nc_N/(n+1)$, $b_1 = 2nc_N / \{n + 1 - 2(2n+1)c_N\}$,

$c_1 = b/\{n - 2nc_N + (1 + n - c_N - 2nc_N)b\}$,

$c_{det} = 4(1+2n)c_N b^2 / \{4n^2 c_N (b + c_N) + (n+1)^2 b^2\}$.

Because the conditions of Eq. (S4.2) permit both cases (ii) and (vi) (Figures 4A and S2, magenta and green), these cases are divided according to whether the population of cooperators (x = 1) is invaded by cheaters (x = 0) or not. Thus, if

$w(1,0) = \{nc(1+b)(1-c_N) - b\}/n < 0$ (i.e. $c < c_{pm} = b/n(1-c_N)(b+1)$), (S4.3)

the resident population is not invaded by cheaters and is stably maintained. Consequently, under the conditions (S4.2) and (S4.3) (i.e. $D(x) > 0$ for $0 \le x \le 1$ and $w(1,0) < 0$), the population evolves to case (ii) "Maximum evolution" (Figures 4A and S2, magenta).

S4.3. Case (vi) Parasitic coexistence by null mutation

By contrast, if conditions (S4.2) and $w(1,0) > 0$ (i.e. $c > c_{pm}$) are both satisfied, cheaters possessing the null mutation can invade a resident population with x = 1, and the population evolves to case (vi) "Parasitic coexistence by null mutation" (i.e. $D(x) > 0$ for $0 \le x \le 1$ and $w(1,0) > 0$) (Figures 4A and S2, green).

S4.4. Case (iii) Intermediate evolution

Intermediate between cases (i) and (ii)/(vi), the parameters satisfy both $D(0) > 0$ and $D(x) < 0$ for some $0 < x < 1$. Thus, singular strategies that satisfy $D(x) = 0$ exist between 0 and 1, of which the smallest (x^*) is described by

$$x^* = (b - nc)/(n+1)bc \quad \text{(for } c_N = 0\text{)} \tag{S4.4a}$$

$$x^* = \left\{c(b + nb - 2nc_N) - \sqrt{det}\right\}/2(2n+1)c_N bc \quad \text{(for } c_N \neq 0\text{).} \tag{S4.4b}$$

This singular strategy x^* is always CS because $D'(x^*) < 0$, indicating that an initial population with $x = 0$ evolves toward x^*. The symbiotic behavior then permits one of two cases depending on the ESS-stability of x^*, namely,

$$E(x^*) = \left\{n(n-1)(b + 2c_N)c - (n+2)\sqrt{det}\right\}/n(2n+1)\,. \tag{S4.5}$$

If $E(x^*) < 0$ (i.e. $c > c_{ESS} = (n+2)^2 c_N b^2 / \left\{3n^2 c_N (b + c_N) + (1 + n + n^2) b^2\right\}$), the singular strategy x^* is both CS and ESS-stable (i.e. $D'(x^*) < 0$ and $E(x^*) < 0$), and the population with x^* is robust to invasion by mutants adopting similar strategies. In addition, it cannot be invaded by cheaters with $x = 0$ imposed by the null mutation, because $w(x^*,0) < 0$ (see Text S4.7). Thus, this condition leads to case (iii) "Intermediate evolution" (i.e. $D(0) > 0$, $D'(x^*) < 0$, and $E(x^*) < 0$) (Figures 4A and S2, blue).

S4.5. Case (iv) Co-dependent coexistence

Contrary to case (iii), if $E(x^*) > 0$ (i.e. $c < c_{ESS}$) is satisfied, the singular strategy x^* is CS but not ESS-stable (i.e. $D'(x^*) < 0$ and $E(x^*) > 0$). This situation is known to cause "evolutionary branching", in which the population of x^* is invaded by nearby mutants and subsequently splits into two subpopulations. Under most of the numerical

conditions investigated in this paper, evolutionary branching leads to stable coexistence of full cooperators ($x = 1$) and full cheaters ($x = 0$). These parameter conditions lead to case (iv) or (v) (Figures 4A and S2, orange and purple), depending on the stability of the monomorphic population of cooperators, determined by the sign of $D(1)$. If $D(1) < 0$ (i.e. $c > c_1$), the population of cooperators cannot maintain their activity against invasion of mutants producing fewer nitrogen resources. This situation leads to case (iv) "Co-dependent coexistence" (i.e. $D(0) > 0$, $D'(x^*) < 0$, $E(x^*) > 0$, and $D(1) < 0$) (Figures 4A and S2, orange).

S4.6. Case (v) Parasitic coexistence by evolutionary branching

Alternatively, if $D(1) > 0$ (i.e. $c < c_1$), a monomorphic population with $x = 1$ should persist without invasion by nearby mutants, leading to case (v) "Parasitic coexistence by evolutionary branching" (i.e. $D(0) > 0$, $D'(x^*) < 0$, $E(x^*) > 0$, and $D(1) > 0$) (Figures 4A and S2, purple).

S4.7. Invasibility of cheating rhizobia in case (iii)

The invasibility of cheating bacteria ($y = 0$) is determined by the sign of

$$w(x,0) = \{nc(1-c_N x)(1+bx) - b\}x/n = -xD(x) + x^2E(x)/2 - c_N bcx^3/n. \qquad \text{(S4.6)}$$

In case (iii), we have $w(x^*,0) < 0$ because the singular strategy x^* is ESS-stable (i.e. $D(x^*) = 0$ and $E(x^*) < 0$). Thus, in case (iii), a population with x^* cannot be invaded by cheaters.

S4.8. Equilibrium proportion of cooperator in cases (iv)–(vi)

In cases (iv)–(vi), emergent cooperators ($x = 1$) and cheaters ($x = 0$) coexist. Now

suppose that the proportions of cooperators and cheaters alter during a cycle of host plants colonization, proliferation in root nodules, and release to the soil (Figure 1A). Let the proportions of cooperators and cheaters before the cycle be p and $q = 1 - p$, respectively. Then the probability that a host plant is colonized by m cooperators and $(n - m)$ cheaters is

$$P(m) = p^m q^{n-m} n! \big/ m!(n-m)! . \qquad \text{(S4.7)}$$

Given that the average nitrogen fixation in the focal plant is $\bar{x} = m/n$, the growth rates of cooperators and cheaters are given by $f_1 = B(m/n)C(1)$ and $f_0 = B(m/n)C(0)$, respectively. Therefore, the expected number of cooperators per host plant after the cycle is

$$g_1 = \sum_{m=0}^{n} P(m) m f_1 = p(1 + c + c_N c)(n + qb + npb) , \qquad \text{(S4.8)}$$

and of the number of cheaters is

$$g_0 = \sum_{m=0}^{n} P(m)(n-m) f_0 = q(n - pb + npb) . \qquad \text{(S4.9)}$$

Then the proportion of post-cycle cooperators is $p' = g_1 \big/ (g_0 + g_1)$. If this proportion is equilibrated, we have

$$p = p' \text{ and } p + q = 1, \qquad \text{(S4.10)}$$

Solving Eq. (S4.10), we obtain the proportion of cooperators at equilibrium as

$$p^* = \{b - (1 - c_N)(b + n)c\} \big/ (n-1)(1 - c_N)bc . \qquad \text{(S4.11)}$$
