# Supplementary material for: Evolutionary Dynamics of Nitrogen Fixation in the Legume–Rhizobia Symbiosis
Source: PLoS One. 2014 Apr 1;9(4):e93670. doi: 10.1371/journal.pone.0093670 (PMC3972148; doi:10.1371/journal.pone.0093670)
Supplement: Text S5 — Systemic and local effects of nitrogen fixation. (PDF) [file pone.0093670.s007.pdf]

# Evolutionary Dynamics of Nitrogen Fixation in the Legume–Rhizobia Symbiosis

Hironori Fujita[1*], Seishiro Aoki[2], Masayoshi Kawaguchi[1,3]

[1] Division of Symbiotic Systems, National Institute for Basic Biology, National Institute for Natural Sciences, Okazaki 444-8585, Japan

[2] Department of General Systems Studies, Graduate School of Arts and Sciences, University of Tokyo, 3-8-1 Komaba, Meguro-ku, Tokyo 153-8902, Japan

[3] Department of Basic Biology, School of Life Science, Graduate University for Advanced Studies (SOKENDAI), Okazaki 444-8585, Japan

[*] E-mail: hfujita@nibb.ac.jp

## Supporting Information

## Text S5. Systemic and local effects of nitrogen fixation

If the fitness is described as the product of the systemic and local effects, we have

$$\varphi_i = S(\bar{x})L(x_i) \quad \text{and} \quad \bar{x} = \sum_{k=1}^{n} x_k \big/ n, \tag{S5.1}$$

where $S(x)$ and $L(x)$ are the systemic and local functions, respectively. The fitness of rare mutants with activity $y$ in a resident population with activity $x$ is then given by

$$f_x(y) = S(\bar{x})L(y) \quad \text{and} \quad \bar{x} = ((n-1)x + y)\big/n. \tag{S5.2}$$

Thus, we have

$$D(x) = \partial f_x(y)\big/\partial y\Big|_{y=x} = S'(x)L(x)\big/n + S(x)L'(x), \tag{S5.3}$$

$$E(x) = \partial^2 f_x(y)\big/\partial y^2\Big|_{y=x} = S''(x)L(x)\big/n^2 + 2S'(x)L'(x)\big/n + S(x)L''(x), \tag{S5.4}$$

$$w(1,0) = f_1(0) - f_1(1) = S(1-1/n)L(0) - S(1)L(1). \tag{S5.5}$$

We then obtain

$$D(x) = L'(x),\ \ D'(x) = L''(x),\ \ E(x) = L''(x), \text{ and } \ w(1,0) = L(0) - L(1) \qquad \text{(S5.6)}$$

in the absence of the systemic effect (i.e. $S(x) = 1$), and

$$D(x) = S'(x)/n,\ \ D'(x) = S''(x)/n,\ \ E(x) = S''(x)/n^2, \ \text{ and } \ w(1,0) = S(1 - 1/n) - S(1) \qquad \text{(S5.7)}$$

in the absence of the local effect (i.e. $L(x) = 1$).

The coexistence of nitrogen-fixing and cheating rhizobia can arise via two pathways: evolutionary branching and null mutation. Coexistence by evolutionary branching requires that $x^*$ is CS but not ESS-stable (i.e. $D'(x^*) < 0$ and $E(x^*) > 0$), where $x^*$ is an evolutionarily stable strategy (i.e. $D(x^*) = 0$). In the cases of $S(x) = 1$ and $L(x) = 1$, this type of coexistence cannot emerge, because the signs of $D'(x)$ and $E(x)$ are always the same (Eqs. (S5.6) and (S5.7)). Conversely, coexistence by null mutation is possible if both $D(x) > 0$ for $0 < x < 1$ and $w(1,0) > 0$ are satisfied. However, if $D(x) > 0$, $L(x)$ or $S(x)$ is an increasing function of $x$, which contradicts $w(1,0) > 0$ (i.e. $L(0) > L(1)$ or $S(1-1/n) > S(1)$, respectively). Consequently, this type of coexistence is also precluded. Therefore, mutualists and cheaters stably exist only if the fitness function includes both systemic and local effects.
